# X-ray fluorescence spectroscopy (XRF) for metallome analysis of herbarium specimens

**DOI:** 10.1186/s13007-022-00958-z

**Published:** 2022-12-19

**Authors:** Imam Purwadi, Lachlan W. Casey, Chris G. Ryan, Peter D. Erskine, Antony van der Ent

**Affiliations:** 1 Centre for Mined Land Rehabilitation, Sustainable Minerals Institute, The University of Queensland, St Lucia, QLD 4072 Australia; 2 Centre for Microscopy and Microanalysis, The University of Queensland, St Lucia, QLD 4072 Australia; 3 CSIRO, Mineral Resources, Clayton South, VIC 3169 Australia; 4 Laboratoire Sols et Environnement, INRAE, Université de Lorraine, Vandœuvre-lès-Nancy cedex, F-54505 France

**Keywords:** Areal density, XRF, Dynamic analysis, Intermediate thickness, Herbarium, Hyperaccumulator

## Abstract

**Background:**

“Herbarium X-ray Fluorescence (XRF) Ionomics” is a new quantitative approach for extracting the elemental concentrations from herbarium specimens using handheld XRF devices. These instruments are principally designed for dense sample material of infinite thickness (such as rock or soil powder), and their built-in algorithms and factory calibrations perform poorly on the thin dry plant leaves encountered in herbaria. While empirical calibrations have been used for ‘correcting’ measured XRF values post hoc, this approach has major shortcomings. As such, a universal independent data analysis pipeline permitting full control and transparency throughout the quantification process is highly desirable. Here we have developed such a pipeline based on Dynamic Analysis as implemented in the GeoPIXE package, employing a Fundamental Parameters approach requiring only a description of the measurement hardware and derivation of the sample areal density, based on a universal standard.

**Results:**

The new pipeline was tested on potassium, calcium, manganese, iron, cobalt, nickel, and zinc concentrations in dry plant leaves. The Dynamic Analysis method can correct for complex X-ray interactions and performs better than both the built-in instrument algorithms and the empirical calibration approach. The new pipeline is also able to identify and quantify elements that are not detected and reported by the device built-in algorithms and provides good estimates of elemental concentrations where empirical calibrations are not straightforward.

**Conclusions:**

The new pipeline for processing XRF data of herbarium specimens has a greater accuracy and is more robust than the device built-in algorithms and empirical calibrations. It also gives access to all elements detected in the XRF spectrum. The new analysis pipeline has made Herbarium XRF approach even more powerful to study the metallome of existing plant collections.

**Supplementary Information:**

The online version contains supplementary material available at 10.1186/s13007-022-00958-z.

## Background

Herbaria around the world are the greatest sources of taxonomic, genetic, and biogeographic information on plants. As stores of well-curated plant material, they also represent an opportunity to gather further information via non-destructive techniques, but this potential remains largely unexplored to date. Recently, we have proposed the “Herbarium X-ray Fluorescence (XRF) Ionomics” approach to quantify elemental concentrations from herbarium-based plant collections [1]. Ionomics is a term referring to the study of the complete ionomic or elemental composition of a plant species [2, 3] and is congruent with the metallome or elementome (sensu [4]) in this context. It requires technologies that enable high-throughput elemental analysis on large numbers of samples to gain insights in how foliar elements correlate to the ecophysiology of different plant species and how they are regulated. The herbarium XRF ionomics approach enables to quantify most elements ranging from aluminium to uranium in less than one minute [5]. This cutting-edge methodology is game-changing for the analysis of element concentrations in plant leaves across phylogenetic lineages [1].

The XRF instrument subjects a sample with a beam of high-energy X-rays generated from a X-ray tube inside the device which causes X-ray fluorescence to occur in the sample [6]. These excited fluorescent X-rays are then recorded and analysed to calculate the relative concentrations of elements present in the sample [7]. The technique has low detection limits for many different elements of the Periodic Table [8]. Compared to wet chemical analysis that involves cumbersome acid-digestion followed by ICP-AES/MS analysis, handheld XRF is a rapid method elemental analysis of plant material, and is therefore appealing to plant scientists when analysing hundreds or even thousands of samples [9–12]. In combination with simple sample grinding, a handheld XRF instrument can achieve an accuracy comparable to digestion-based methods with inductively coupled plasma atomic emission spectroscopy (ICP-AES) and surpases the latter for refractory elements, such as silicon or chromium, because of incomplete solubilization during the digestion process [10]. Many studies have developed a single element calibrations for plant matrices [9, 11], but a new calibration is still required for quantifying an element that is not included in the existing calibration dataset [13].

Recently, handheld XRF instrumentation has been repurposed to determine the totality of metal and metalloid concentrations of herbarium specimens [1]. The focus has been largely on identifying hyperaccumulator plants, which are plants that can naturally accumulate > 10,000 µg g^−1^ of manganese (Mn), > 3000 µg g^−1^ of zinc (Zn), > 1000 µg g^−1^ of nickel (Ni), arsenic (As), Rare Earth Elements (REEs), > 300 µg g^−1^ of cobalt (Co), copper (Cu), > 100 µg g^−1^ cadmium (Cd), thallium (Tl), or selenium (Se) in their dry weight foliar mattter [14–16]. Discovery of hyperaccumulator plant species is imperative because they can be used for phytoremediation, phytomining and mineral exploration applications [14, 17] and are frequently in danger of extinction due to mining activities as most grow naturally on metalliferous soils that are mining targets [18–20].

When performing XRF scanning of herbarium specimens, only 30 s is needed to obtain quantitative data [5], it does not cause any damage the herbarium specimens [1], and it reduces the resources spent on collecting specimens [21], thereby enabling high throughput systematic scanning of herbarium collections orginating from around the world. The herbarium XRF approach was first conducted at the Forest Research Centre (Sabah Forestry Departement) in Sabah (Malaysia) on the genus *Antidesma*, and a critically endangered Ni hyperaccumulator plant species was identified [22]. A follow-up study led to the discovery of 28 Ni hyperaccumulator plant species, 12 Co hyperaccumulator plant species, and 51 Mn hyperaccumulator plant species from the 7300 specimens analyzed [5]. No hyperaccumulator plant species were known from Papua New Guinea before scanning of herbarium specimens of native plants from that country identified 10 Zn hyperaccumulator plant species, 8 Mn hyperaccumulator plant species, 1 Ni hyperaccumulator plant species [23]. Furthermore, herbarium XRF scanning was performed in New Caledonia on 11,200 specimens and numerous new hyperaccumulator plant species were identified: 63 Mn hyperaccumulator plant species, 5 Co hyperaccumulator plant species, 34 Ni hyperaccumulator plant species, and 4 Zn hyperaccumulator plant species [24]. In Central America, herbarium specimens of *Psychotria costivenia* and *P. grandis* were identified to be Ni hyperaccumulator plant species using XRF scanning of herbarium specimens [25].

**Fig. 1:**
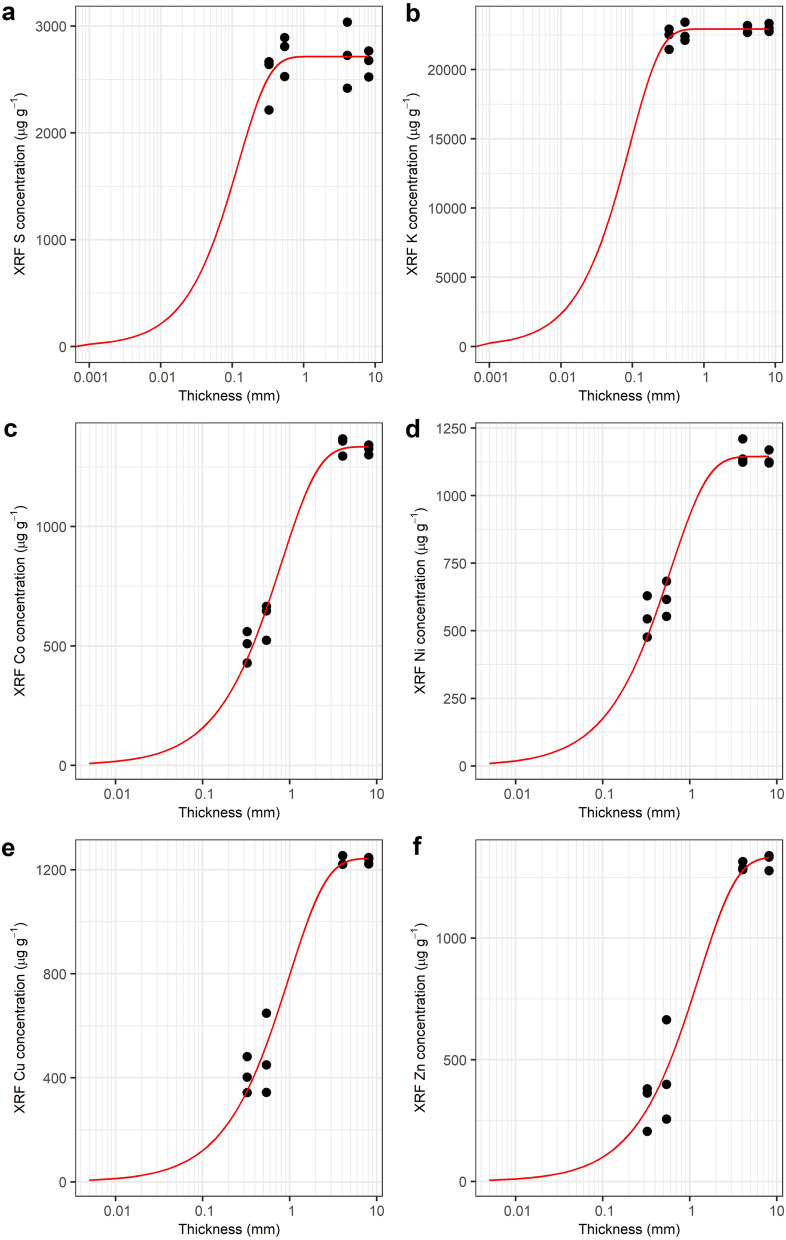
The effects of thickness on the XRF readings. Red lines are a model fitted into the reported concentrations (black dots) following Eq. (1). The samples (black dots) are constant in concentration but vary in thickness. The model reaches a plateau at a certain thickness that signifies the escape depth or critical thickness of the respective elements and shifts towards the right direction of the x-axis as the atomic number of the elements gets higher. Small variations in the sulphur and potassium XRF results are due to random noise and indicate that the samples are considered as thick samples for XRF. The underlying data used is provided in Additional file 1

Three different approaches are usually employed to convert the measured intensities of fluorescence X-rays in the XRF spectrum to elemental concentrations in the sample, namely Fundamental Parameters, Compton normalization, and Empirical Calibration [26, 27]. A relationship between measured fluorescence peak intensities and the concentration of elements in the sample is theoretically expressed as a complex physics equation [28]. Fundamental Parameters is an approach that solves the equation by providing all parameters required by the equation [29]. In the XRF quantification process, a sample is classified into thin sample, intermediate sample, or thick sample. For thin samples, both enhancement and absorption effects are minimized, and the analyte signals follow a linear function of thickness [30]. Thick samples exceed a saturation thickness above which the intensity of the characteristic lines is constant and independent of sample thickness [30], as illustrated by Fig. 1a, b Between these extremes, the fluorescence intensities vary with thickness (Fig. 1c–f). The handheld XRF instrument is usually calibrated based on Fundamental Parameters and assumes the sample to meet the requirement of an infinitely thick sample [31]. Because of that, it requires samples to have a certain thickness and to be homogenized (i.e., by grinding, mixing, and compacting) [21]. Failing to fulfil these requirements will cause systematic errors [26]. The Compton normalization technique normalizes the intensities of fluorescence peaks to the intensity of the Compton scatter [32]. However, Compton normalization may not correct all matrix effects [33]. In the case of herbarium XRF scanning, empirical calibrations have been employed to ‘correct’ the raw XRF-instrument generated values by measuring a set of dry leaves and establishing a regression model between these XRF values and ICP-AES measured values of these same samples [22–25]. Empirical calibration offers a simple and practical solution to correct the XRF result without any information on the properties of the instrument and sample required, but it is prone to errors [13] because it expects the calibration standards to have the exact same matrix as the actual samples which may vary in thickness, density, and composition [34]. Compared to the empirical calibration, Fundamental Parameters approaches, such as Dynamic Analysis implemented in GeoPIXE software, do not require standards, and can be used for predicting the concentration of any element, while the empirical method needs a set of standards for each different element and sample type [29, 35–37]. However, the major shortcoming of the Fundamental Parameters approach is that prior knowledge of the sample areal density (g/cm^2^) is required [38, 39]. Many approaches to simultaneously determine the areal density of a sample and its elemental composition have been proposed, including adding an internal standard [40] or combining XRF with other techniques [41], however none of these methods is compatible with herbarium XRF measurements, which must be fully non-destructive. Alternatively, the areal density of a sample can also be determined using the emission-transmission technique [42], which requires three measurements: that of a herbarium specimen, that of a substrate (e.g. a pure thick element), and that of a herbarium specimen on top of that substrate [35, 42].

**Fig. 2:**
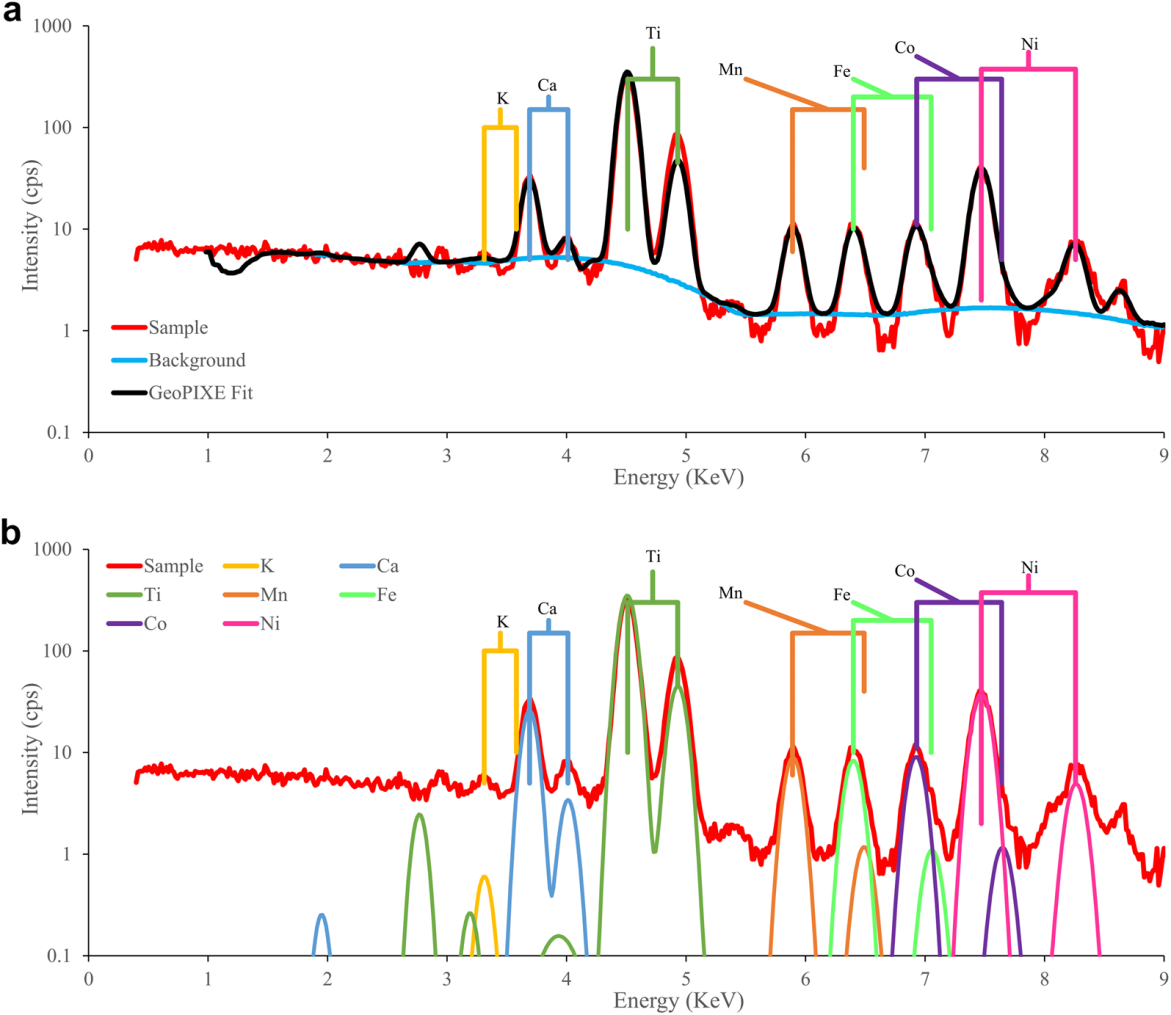
Fitted XRF spectrum of a sample processed in GeoPIXE in a non-linear least-square fit (black trace) to an XRF spectrum of *Glochidion sericeum* (red trace) in **a**. *Glochidion sericeum* is a Ni-Co hyperaccumulator plant. The fitted spectrum is deconvolved into single fluorescence element peaks **b**. Note that Dynamic Analysis can deconvolve overlapping peaks such as the Fe Kβ peak from the Co Kα peak, and the Mn Kβ peak from the Fe Kα peak

Dynamic Analysis (DA), which is based on Fundamental Parameters, has been developed for nuclear microprobe analysis and was extended for use in synchrotron-based XRF analysis [43, 44]. It first uses a non-linear least-squares approach to fit the non-linear parameters, such as peak width, energy calibration and tailing in the spectrum, and then performs a further linear fit iteration with the non-linear parameters fixed in order to construct a transform matrix (the DA matrix). In contrast to early Fundamental Parameter approaches [45], this transform is applied directly to spectral data to deconvolute the spectrum into the fluorescence components for each element [46]. As shown in Fig. 2, use of the DA transform then acts like a linear LS fit. One of the many benefits of this approach, aside from fast execution, is that it can deal with complex spectra with numerous line overlaps, for example, the known interference between the Co Kα_1_ line (at 6.9303 keV) and the Fe Kβ_1_ line (at 7.058 keV) which makes it difficult to resolve Co if Fe is present at high concentrations. Figure 2 shows an XRF spectrum of the Ni-Co hyperaccumulator *Glochidion sericeum*, and as can be seen from Fig. 2b, Dynamic Analysis can separate the line overlaps successfully. As such, Dynamic Analysis is a powerful alternative to the built-in algorithms of a handheld XRF instrument while providing complete control over the input parameters, as opposed to the ‘blackbox’ proprietary software used by XRF instrument manufacturers. GeoPIXE is a software package that implements Dynamic Analysis, and provided that all of the required information for the Fundamental Parameters can be obtained, the calculated sample elemental concentrations are fully quantitative [47].

This article presents and validates a new data analysis pipeline (based on Dynamic Analysis) to process the raw data obtained from handheld XRF instruments for the measurement of the ionome/metallome of herbarium specimens. This pipeline is aimed to be universal permitting full control and transparency throughout the quantification process independent of the XRF instrument make or model. Furthermore, we will combine this pipeline with a new method based on the emission-transmission technique to determine the areal density of herbarium specimens. The new pipeline will be compared to the results obtained from the XRF instrument built-in algorithms and to the empirical calibration approach.

## Results

The Dynamic Analysis approach requires a complete description of both the instrument and the sample. However, many properties of the instrument are proprietary and are often undisclosed by the manufacturers. However, even with incomplete knowledge of the XRF system, it is possible to refine the model using simple standards. In this case, three 100 nm thin films [Ti, gold (Au), and tin (Sn)] with well-spaced lines covering multiple energy regions, were used to refine the instrument’s sensitivity and absorption characteristics. The thin films were measured by the handheld XRF device used in this study, and the raw spectra of the thin films were processed in GeoPIXE. Parameters related to the instrument, such as the X-ray source, distance, angle, filter, and detector were based on available information as well as physical inspection of the device, and then refined using the thin-film standard measurement data to yield results close to the expected 100% concentration for each element. Table 1 shows the final concentrations of Ti, Au, and Sn obtained in this study, and the used parameters can be found in Additional file 1.

**Table 1:** The concentration of pure thin films obtained after deducing the parameters of Thermo Fisher Scientific Niton XL3t 950 GOLDD

Element	Ti	Au		Sn	
Fluorescence line	K	L	M	K	L
Concentration (%)	104	98	98	100	98

The instrument-related parameters adjusted to produce this number can be found in Additional file 1

**Fig. 3:**
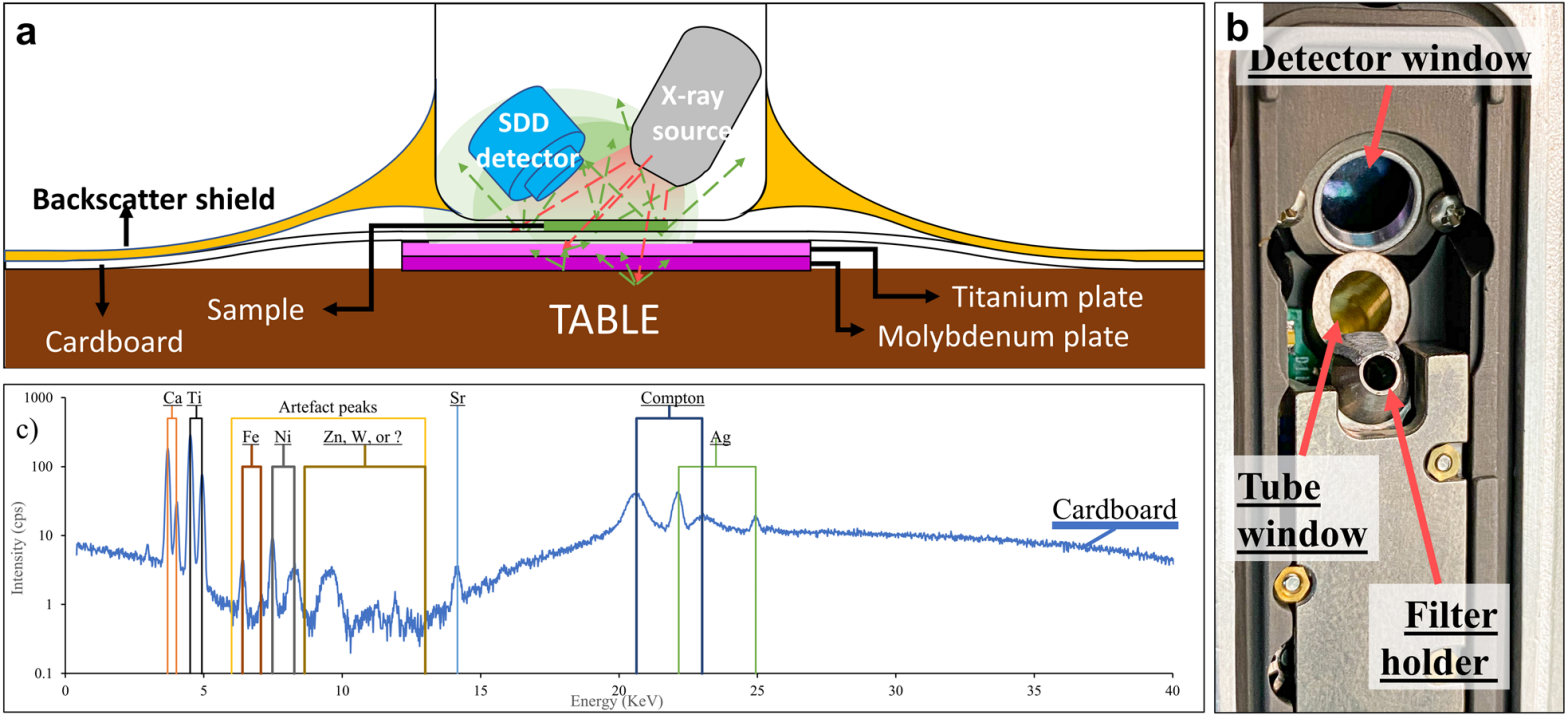
A schematic diagram illustrating the typical herbarium XRF measurement setup in **a**. **b** A photo shows the front view of the Thermo Fisher Scientific Niton XL3t 950 GOLDD+ with the face plate removed, revealing the detector window, tube window, and filter holder. **c** Fluorescence artefact peaks which spectrally coincides with the position of Fe, Ni, Zn, and unknown peaks are observed in the XRF spectrum of the cardboard measured according to the setup shown in **a**. No first-row transition metal was detected in the cardboard except for Fe which is 69 µg g^−1^ based on ICP-AES analysis

Herbarium specimens are typically affixed to a cardboard backing, and this must be dealt with during the measurement. The composition and thickness of this cardboard may vary from herbarium to herbarium. An example of such a cardboard backing was measured while placing a 2 mm thick pure Ti plate underneath. Artefact peaks were observed between 6 and 13 keV as shown in Fig. 3c. ICP-AES analysis confirms that the cardboard contains Ca at 110, 115 µgg^−1^, and this is the likely source of the strong Ca peak. The ICP-AES results also confirm that the cardboard contains Fe, but only at 69 µg g^−1^, which is below the stated detection limit of the handheld device. To investigate the origin of these peaks, the 'pure' excitation spectrum of the device was obtained by triggering the X-ray tube of the handheld device into the SDD detector of a benchtop micro XRF instrument (IXRF ATLAS X). This confirmed the composition of the anode (Ag) and showed that the artefact peaks were not present in the incident beam itself, but most likely originate from secondary fluorescence from elements (such as metal alloy components) in the chamber of the handheld spectrometer and/or diffraction effects (which cannot be determined easily).

**Fig. 4:**
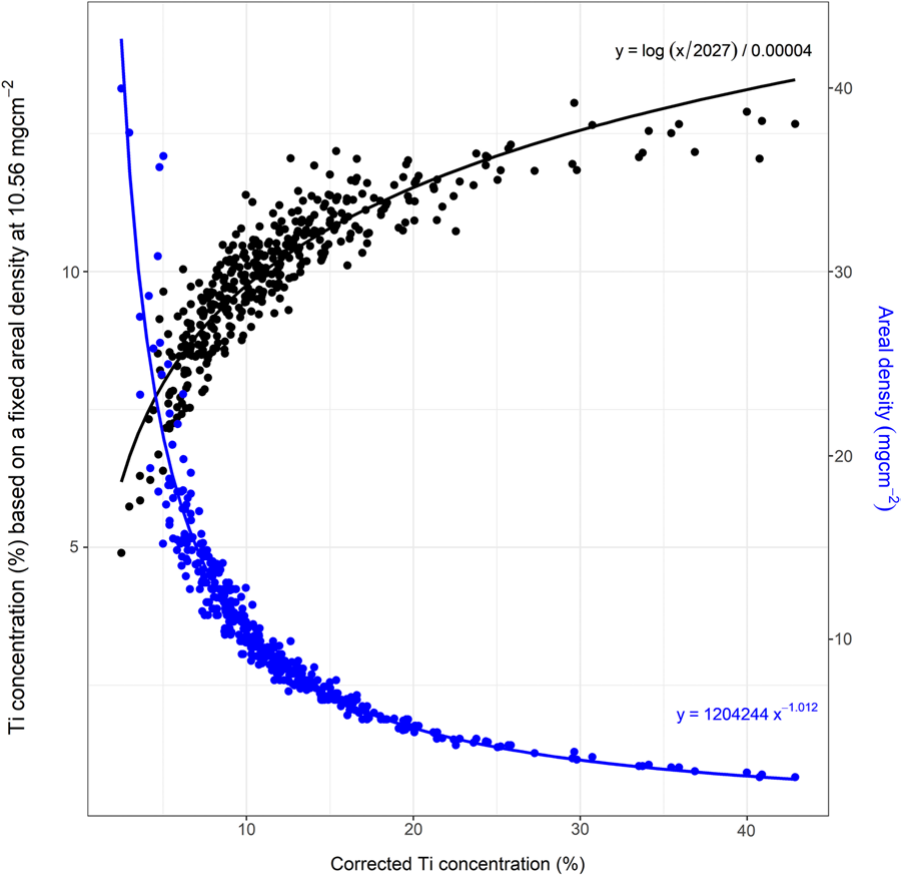
Models for predicting the areal density of unknown samples, using the degree of attenuation of the signal from a pure Ti plate underneath each sample. First, a fixed areal density is used to produce an initial Ti concentration (black line). This Ti concentration is then used to predict the areal density of the specific sample via (blue line). The initial areal density used to produce dataset on the x-axis of (black line) is 10.56 mg/cm^2^ which is the mean measured areal density of the training dataset

**Fig. 5:**
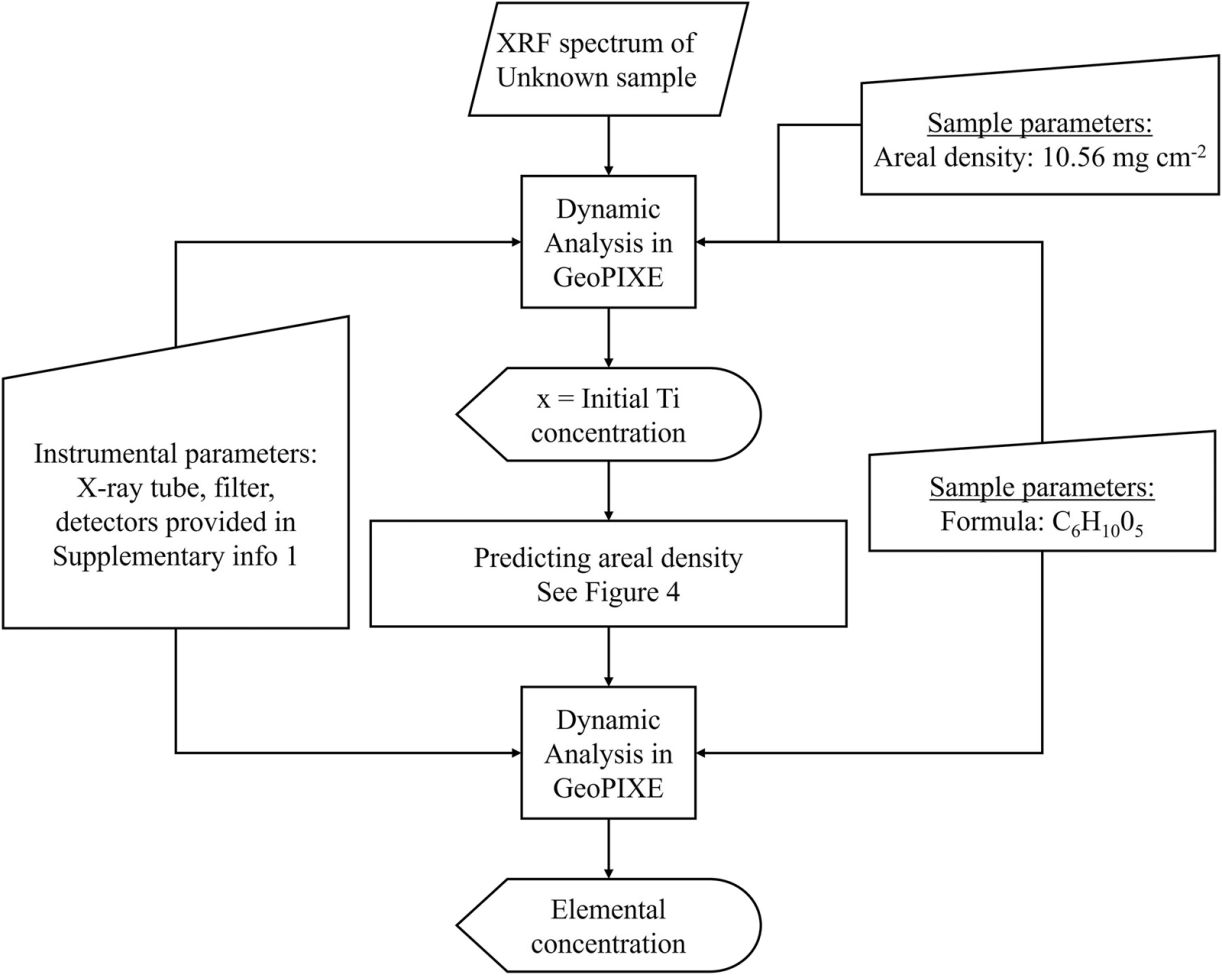
The flowchart of the analysis pipeline for determining the areal density and elemental concentrations based on the Dynamic Analysis approach

To generate a training and testing dataset, a total of leaves from 588 'normal' plants and hyperaccumulator plants were cut into 6 mm diameter discs and measured using the handheld XRF instrument. Empirical models of the areal density were established based on Ti fluorescence radiation originating from the Ti plate under the sample. Two models were generated. The first model is shown in Fig. 4 (black line) and generated by fitting an exponential model into a scatterplot between the Ti concentrations produced by giving the areal density values of 10.56 mg/cm^2^ (mean areal density of all training dataset) to all XRF spectra and the apparent Ti concentrations produced using the measured areal density for each corresponding sample. The measured areal density was obtained by dividing the mass of each sample with the size of 6 mm diameter circle. The second model, as shown in Fig. 4 (blue line), is produced by fitting an empirical model into a scatterplot between the apparent Ti concentration produced by using the measured areal density of each corresponding against the values of the measured areal density. By using the two models subsequently, the mean absolute error of the predicted areal density is 3.19 mg/cm^2^ for training dataset and 3.97 mg/cm^2^ for the testing dataset. Figure 5 shows the flowchart of the procedure used in this study to quantify elemental concentrations in GeoPIXE. The composition of (herbarium) plant leaves was assumed to be that of cellulose (C_6_H_10_O_5_). Note that the herbarium cardboard is considered to be part of the the actual plant leaf for the purpose of the XRF analysis, but given that the cardboard is usually 'clean' for all elements of interest this has no further relevance [13].

**Fig. 6:**
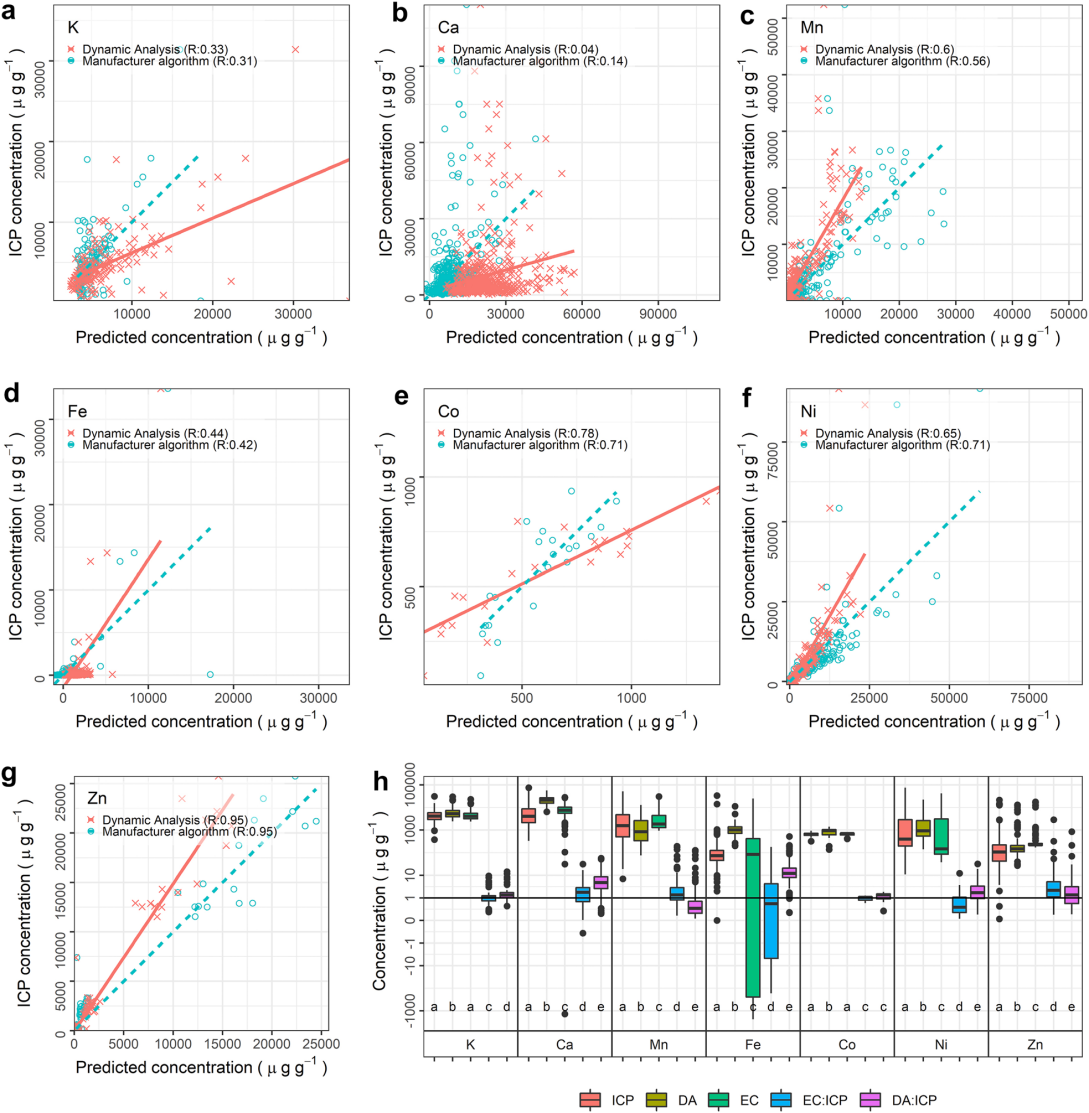
Results of fitting against training datasets. **a**–**g** Scatterplots comparing the empirical calibration and the Dynamic Analysis approaches against ICP-AES results. **h** Box plots in showing results for all methods on the training datasets, together with ICP-AES results (purple). Letter-codes represent the results of a Kruskal–Wallis post hoc test identifying dissimilarity—matching letters indicate that those populations do not significantly differ. DA, ICP, EC in the legends refer to the Dynamic Analysis with the measured areal density, ICP-AES, and empirical calibrations, respectively

**Fig. 7:**
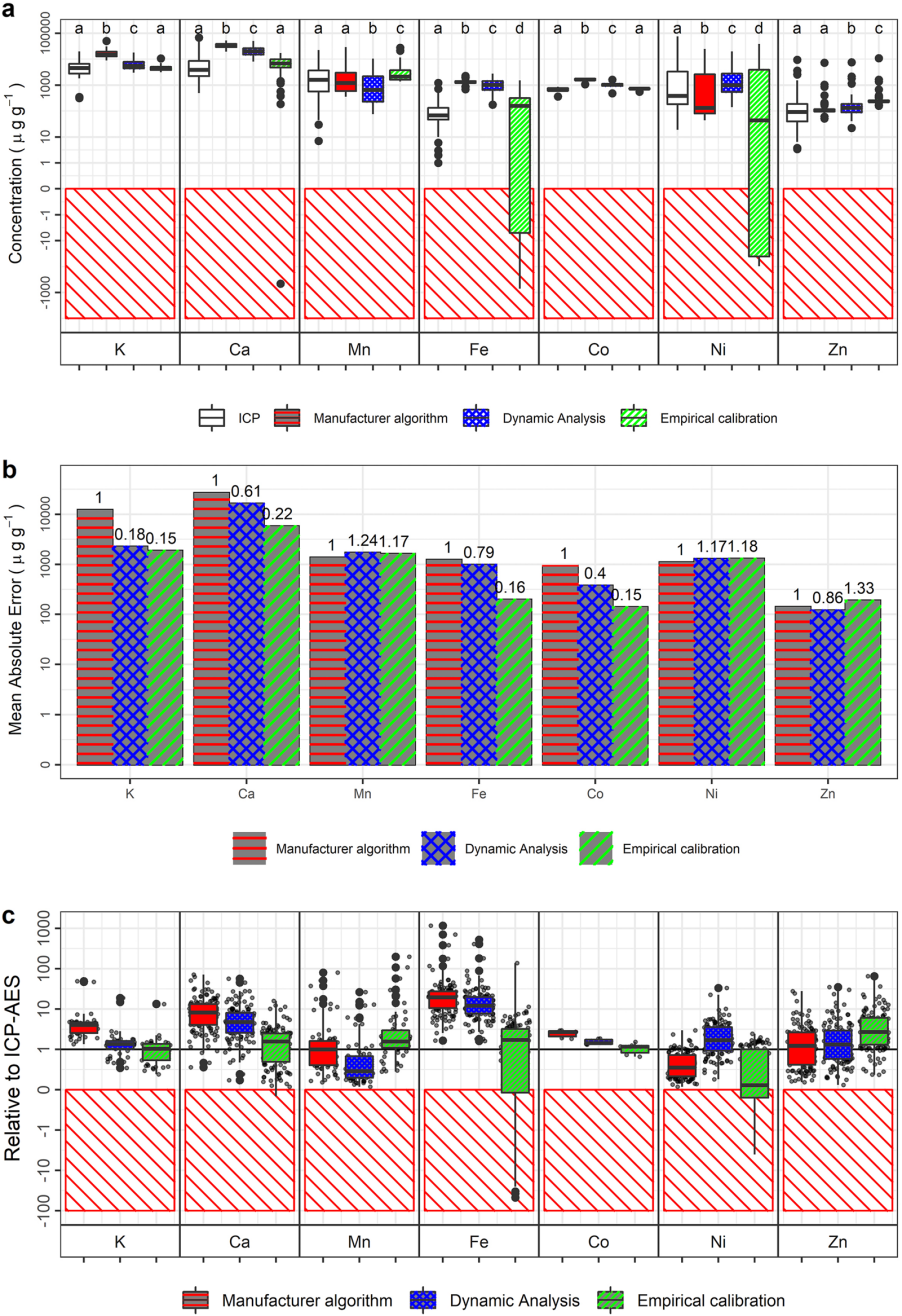
**a** Full elemental concentration results for all approaches, including ICP-AES, shown as box-plots. Letter-codes represent the results of a Kruskal–Wallis post hoc test identifying dissimilarity; matching letters indicate that those populations do not significantly differ. **b** Elemental concentrations relative to ICP-AES concentrations. Small black dots represent the concentrations of each sample. **c** Absolute errors relative to ICP-AES. Red hatches indicate non physical/negative values

These training samples were also used to create empirical calibration models. The 6 mm rounds were analysed by ICP-AES to determine absolute elemental concentrations. Then, a regression model between the ICP-AES results and the results of the instrument’s built-in algorithm was developed. Figure 6 shows elemental concentrations from the training dataset, with the ICP-AES results plotted against both the empirical calibration and the Dynamic Analysis. Kruskal–Wallis tests were performed to assess whether the predicted results are similar or not to ICP-AES results, and the results indicated that only the concentrations of K and Co predicted by empirical calibration are statistically similar to the ICP-AES results. The performance of Dynamic Analysis was also compared to the built-in algorithm and empirical calibration in predicting the elemental concentration of the testing dataset as shown in Fig. 7. Empirical calibration and Dynamic Analysis performed better than the built-in algorithm with errors up to 15% of the built-in algorithm errors in predicting potassium (K), calcium (Ca), iron (Fe), Co, and Zn concentrations. However, errors generated by empirical calibration and Dynamic Analysis methods were comparable or worse with 30% more errors than the built-in algorithm in predicting the concentrations of Mn and Ni (Fig. 7b). Also, from Fig. 7c it can be seen that the empirical calibration generates negative values for the concentrations of Ca, Fe, and Ni (consistent with the results based on the training dataset, see Fig. 6h).

**Fig. 8:**
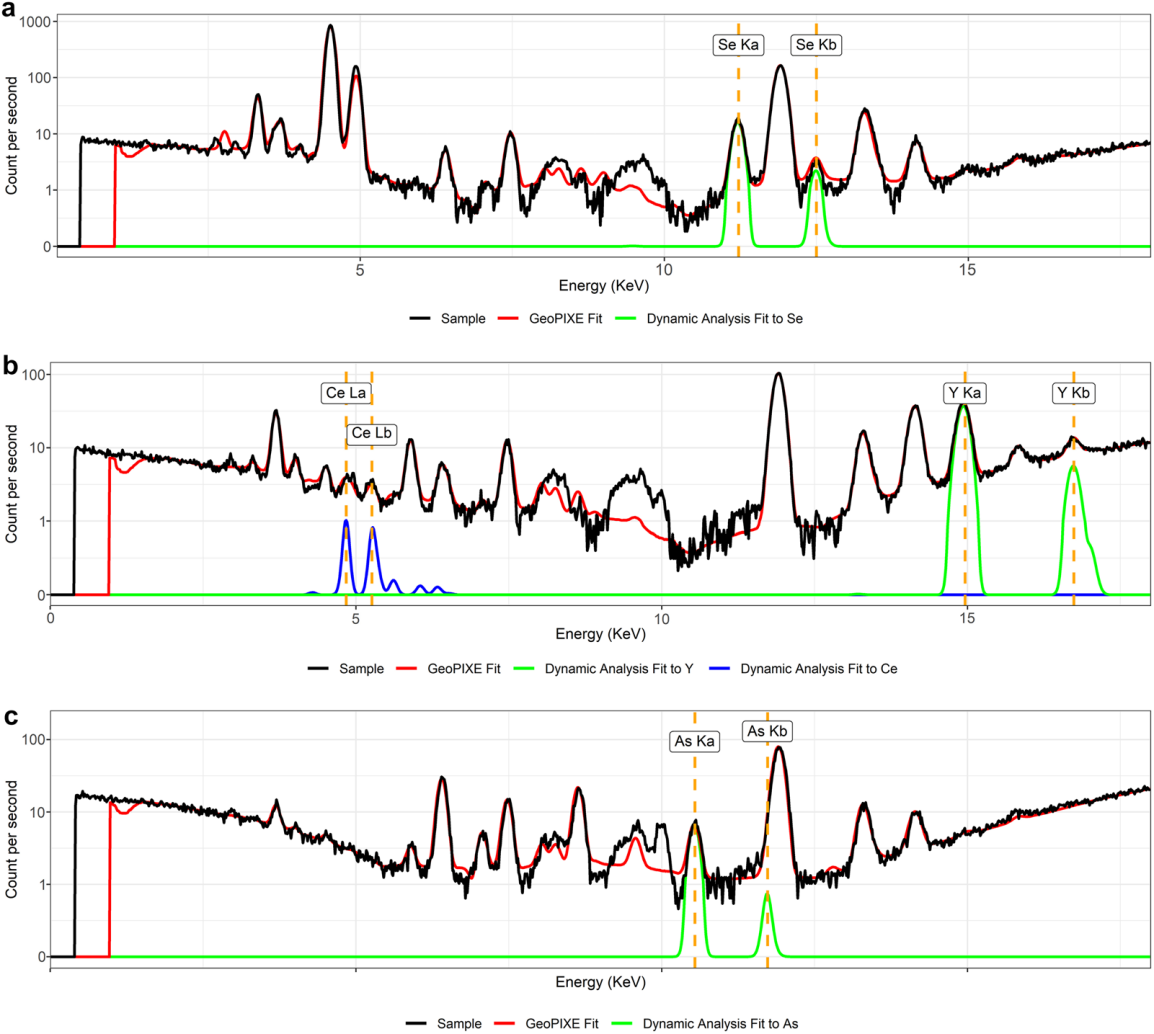
Line plots showing the spectra of herbarium specimens (in black traces) detected to have anomalous concentrations of selenium (**a**), yttrium (**b**), and arsenic (**c**)

Unlike the empirical calibration that requires a calibration line for each element, the Fundamental Parameter approach, as implemented in Dynamic Analysis, uses a global calibration which can then be applied to all elements, provided the lines used for the calibration are well-spaced. To demonstrate this, we used our built model to reprocess a total of 30,013 raw XRF spectra obtained in previous studies in Sabah (Malaysia) (7377 specimens) [23], New Caledonia (11,200 specimens) [24], and Queensland (11,436 specimens). The Dynamic Analysis approach identified several herbarium specimens with high concentrations of selenium, arsenic, and yttrium, which were not identified by the built-in algorithm and the empirical calibration (Fig. 8) and provides a good estimate of their relative concentrations in the specimens.

**Fig. 9:**
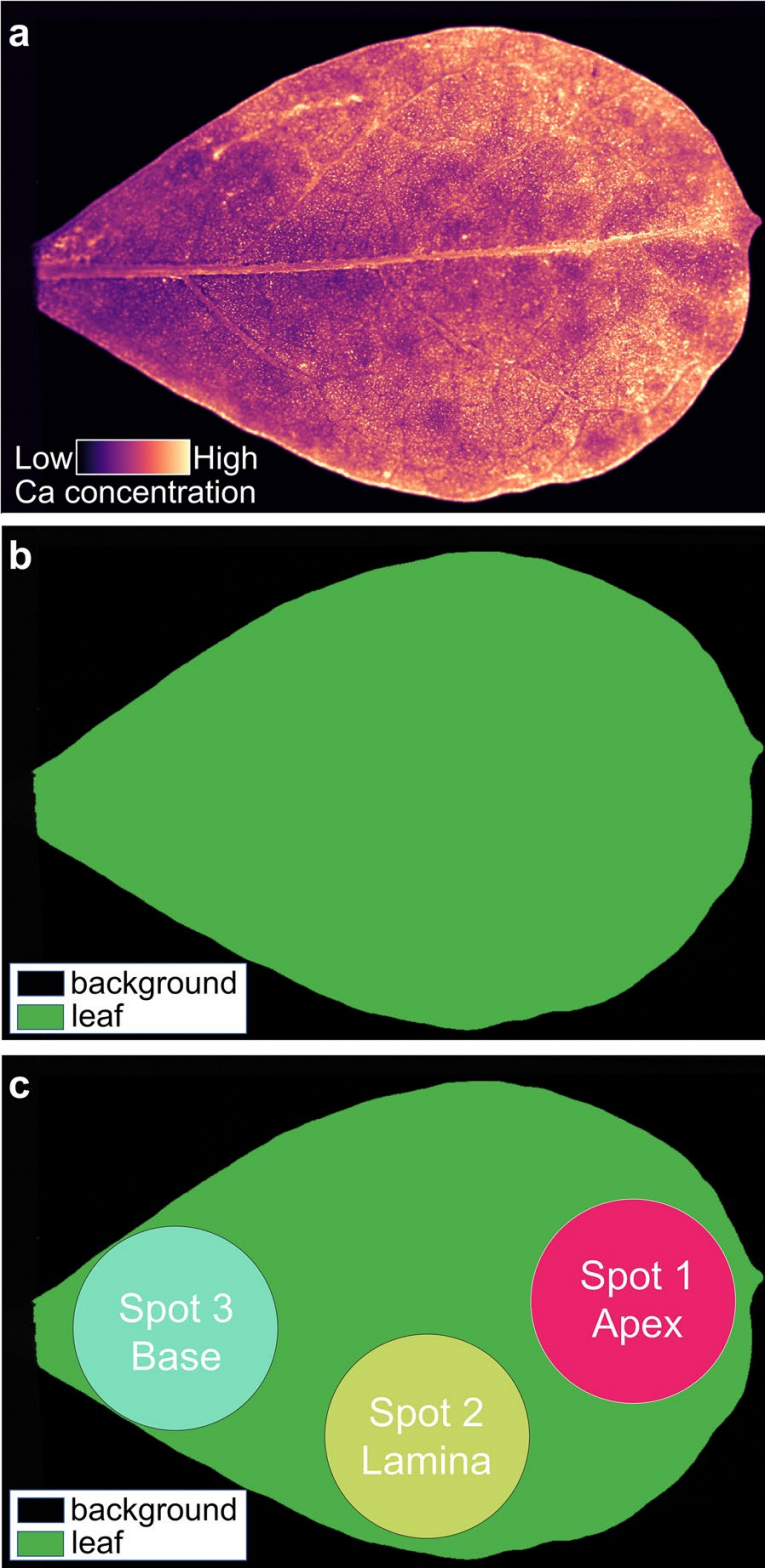
An elemental concentration image generated by GeoPIXE after processing a µXRF image (**a**) was classified into background and leaf (black and green in **b**). Three spots were drawn at the apex, lamina, and base of the leaf part. The average concentration of the whole leave (green in **b**) was compared to the average concentration of the three spots and their combinations. The results are shown in Table 2

The heterogeneity within the specimens reduces the accuracy of the XRF measurement, and averaging multiple measurements is recommended to increase the accuracy [48]. Twenty three intact leaves were analyzed with µXRF analysis to produce high-resolution elemental maps of the leaves showing spatial variation in elemental concentrations. The average elemental concentrations of the whole leaves were compared to the concentrations of subset areas to assess the effect of the number of measurements and of the location on accuracy. In each leaf, three subset areas with a shape of a 6 mm-diameter circle (matching the XRF measurement area of the leaf discs used earlier) were drawn around the apex (1), lamina (2), and base (3) as shown in Fig. 9. Mean absolute percentage error concentrations between the whole leaf and the subsets or their combinations are calculated and tabulated in Table 2. The average of three measurement at the apex, lamina, and base consistently attains the best accuracy ~ 5% or lower with an average of 2.3% across eight different elements (K, Ca, Mn, Fe, Co, Ni, Zn, and Se). Only one measurement at apex or base generates errors more than 20% for K and around 9% on average. Meanwhile, one measurement at lamina performs twice better than one measurement at the apex or base (4.3% compared to 9.5% and 9.1%, respectively). This number is comparable or better than two combinations of the three (apex:lamina:1–2 at 4.2%, apex-base:1–3 at 3.7%, lamina-base:2–3 at 5.6%).

**Table 2:** Mean absolute percentage error of mean concentrations of a subset 6 mm diameter circle around the apex (1), lamina (2), and base (3) and their combinations compared to the mean concentrations of the whole leaves

Combination	K (%)	Ca (%)	Mn (%)	Fe (%)	Co (%)	Ni (%)	Zn (%)	Se (%)	Average (%)
1	22.8	13.1	8.3	5.7	7.3	10.2	5.3	3.5	9.5
2	9.3	6.1	3.7	2.3	3.1	3.9	2.8	2.9	4.3
3	24.7	13.7	12.4	7.0	2.9	4.1	5	2.8	9.1
1,2	11.1	5.6	3.2	2.8	2.3	3.4	2.8	2.8	4.2
1,3	8.1	4.8	2.2	2.7	2.3	4.3	2.2	3	3.7
2,3	14.2	8.5	7.0	3.6	2.8	3.2	3.1	2.3	5.6
1,2,3	5.2	3.1	2.1	1.2	0.7	1.7	1.9	2.7	2.3

## Discussion

Three 100 nm films made of pure Ti, Au, and Sn metals deposited on Kapton film were used to deduce the instrument-specific parameters, related to the detector (diameter, distance, thickness, size, and tilt angle), filters (material and thickness), and source (filter material and thickness). It was achieved by adjusting the parameters to yield Ti, Au, and Sn concentration as close as possible to 100%. After trial and error, the optimum results are shown in Table 1 with errors less than 5% for all fluorescence lines. During inspection of the raw spectra, artefact peaks were observed in the vicinity of Ti K line, Au M and L lines, and Sn L line peaks (Fig. 3b). To investigate whether the origin of these artefact peaks are from the built-in X-ray source, the X-rays emitted by the X-ray source were measured with an external XRF detector without any filter between the X-ray source and the detector except for the air path. The results show that there are no artefact peaks in the obtained spectrum, thus eliminating the possibility of the artefact peaks arising from the X-ray source.

Simple background subtraction was performed to remove these artefact peaks. However, the Dynamic Analysis algorithm did not recognize the subtracted spectra because several channels or bins of the subtracted spectrum have a negative value. Due to the complexity of this issue, no functionality to remove the artefact peaks is available yet in GeoPIXE. However, it may be possible to implement a facility to make a fake “element” with peak energies, widths and relative intensities that can be specified to address this issue. In the case of spectrum range in the vicinity of Sn K line peaks, no artefact peaks were present, and the reported concentration derived from Sn K line peaks was 100%. As Fundamental Parameters requires all parameters included in the calculation, failing to correct the artefact peaks may hamper the Dynamic Analysis approach in obtaining 100% accuracy.

The built-in algorithm performs best at predicting the concentrations of Mn and Ni with the errors of the empirical calibration and Dynamic Analysis up to 24% more than the built-in algorithm errors (Fig. 7). Considering the positions of Mn and Ni fluorescence peaks, the artefact peaks from the instrument may be the reason for poor performance of both the empirical calibration and the built-in algorithm as they are in the same vicinity. For other elements, the higher errors produced by the built-in algorithm are not surprising: the manufacturer algorithms are designed for hard/dense materials (such as rocks and metal alloys) which are very different in composition, density and thickness compared to herbarium specimens [13]. The empirical calibration produces negative values for Ca, Fe, and Ni concentrations, making it unreliable prediction method for this study. These negative values could be due to errors inherited from the built-in algorithm because the concentrations reported by the instrument are not corrected for the matrix effects (absorption and enhancement) and thickness variations. Furthermore, the Dynamic Analysis approach shows its capability to compensate for the absorption and enhancement effects as shown in Fig. 6f, g where the predicted concentrations are closer to the regression line. This result signifies that the proposed approach in predicting areal density of unknown samples performs as well as Dynamic Analysis with the calculated areal density.

The leaf discs used in this study are similar to intact herbarium plant leaves and as a result, sample heterogeneity and thickness affect the accuracy of the measurement. The use of the emission-transmission technique helps to slightly improve the R^2^ values (Fig. 6), but the effects of sample heterogeneity (elemental distributions and thickness variations) are still not fully corrected, and this affects especially light elements (K and Ca) more because of their vertical elemental distributions within the leaf. The leaf discs prepared for this study consist of not only lamina but also veins and midribs. The thick leaf structures (veins and midribs) contribute more signals than the thin leaf structure (lamina), as illustrated in Fig. 1. It is also possible that an element is more concentrated in the thick leaf structure, thereby producing a stronger signal. A sheet of cardboard paper was placed underneath the leaf discs for the measurement, mimicking the way herbarium specimens are mounted on cardboard sheets. The ICP-AES results show that the paper contains appreciable Ca and Fe. The leaf disc may absorb most of this Ca and Fe fluorescence coming from the cardboard, but a portion will egress through the leaf disc as a dry leaf is not infinitely thick for Ca and Fe [13]. This can be seen in Fig. 6b, d. The ICP-AES analysis reports low values but, the built-in and Dynamic Analysis algorithms report high values for both elements because the algorithms quantifies the signal from the cardboard.

The throughput of the XRF scanning of herbarium specimens is a trade-off between speed, accuracy and sensivity. When targeting to obtain datasets of tens of thousands of herbarium specimens, obtaining an error of less than 5% is acceptable. Based on Table 2, overall errors less than 5% can be achieved by measuring once at the lamina (4.3%), an average of two measurements at the apex and lamina (4.2%), or at the apex and base (3.5%) or an average of three measurements at the apex, base, and lamina (2.3%). Measuring twice will slightly reduce the errors up to 0.8% compared to measuring only once at lamina, and may be practical when the number of specimens are not that great (e.g., less 1000 specimens). Meanwhile, three measurements has errors at 2.3%, but takes three times longer. Considering that the herbarium ionomics approach aims to scan millions of specimens around the world, sacrificing 2% accuracy from 2.3 to 4.3% is in our view acceptable in return to reduce the time of the measurement by 67%.

## Conclusions

Herbarium XRF Ionomics is leading the way for increased discovery of hyperaccumulator plant species and the emerging field of studying the plant metallome/elementome. The Herbarium XRF scanning approach is another value-adding proposition for maintaining collections in global herbaria [1]. We developed a new method to determine the areal density of a dry leaf by combining the emission-transmission method with Dynamic Analysis, and by using this approach, this new pipeline outperforms the built-in algorithms. This pipeline is universal and transferable to other XRF istruments from various manufacturers, provided the relevant instrument-specific parameters are known. The XRF instrument built-in algorithms are not customizable and are unable to report some elements. The GeoPIXE-based pipeline focused on the first-row transition metal and in future may be tested on solving the overlapping fluorescence lines of high Z elements with low Z elements, thus helping in discovering more types of hyperaccumulators plants. This is particularly relevant for rare earth elements, as the XRF instrument cannot excite the Kα-lines of the REEs because their absorption edges too high, and only the L-lines will hence be excited. However, the L-lines of the REEs range from 4.64 to 8.71 keV and overlap with the Kα-lines of the first-row transition metals (4.51–8.63 keV), which makes spectral fitting very challenging. Currently, GeoPIXE caters mainly for synchrotron and desktop XRF users, but given an increase in the use of handheld XRF instrumentation, further development of GeoPIXE is highly recommended to facilitate the specific requirements for handheld XRF users, for example, to address artefact line corrections and data handling.

Thus far, the potential to unlock foliar elemental information from herbarium collections has not been fully realised. Apart from focussing on trace element (hyper)accumulation, the full metallome profile could be used to infer plant origin and adaptations of traits measurable with XRF. For example, to probe the incidence of salt tolerance/halophytes on constructed phylogenies (using rubidium and strontium as proxies), to determine the incidence of carboxylate-producing cluster-roots (using yttrium as a proxy), or to study the evolution of selenium (hyper)accumulation in the giant *Astragalus* genus. The use a universal independent data analysis pipeline that offers full control the quantification process, as proposed in this study, will enable future investigations along these lines of inquiry.

## Methods

### Handheld X-ray fluorescence spectrometer

The handheld XRF insutrument (Thermo Fisher Scientific Niton XL3t 950 GOLDD+) has a built-in miniaturized X-ray Ag tube operated at 6–50 kV and with 0–200 µA max current. The instrument was set to the pre-configuration ‘Soils Mode’ with the ‘Main filter’ for all measurements. This mode incorporates Compton Normalisation to convert the measured XRF intensities into elemental concentrations. A total of 588 hyperaccumulator plant leaves was randomly selected from our sample storage collection. Each of 588 leaf samples was cut to a 6 mm diameter disc and put in a dehydrating oven for 48 h at 60 °C. Each leaf disc sample was XRF scanned for 60 s on top of 250 gsm cardboard, a 2 mm thick pure (~ 99.99%) titanium (Ti) plate, and a 2 mm thick pure molybdenum (~ 99.99%) plate. The Ti plate was placed on top of the Mo plate to absorb the Mo fluorescence. The pure Ti plate was chosen as the substrate in determining the areal density because the concentration of Ti in plant is universally low < 34 µg g^−1^ [49, 50] and the escape depth of Ti fluorescence is around 3500 µm in the leaf matrix [13]. The cardboard was placed in between the sample and the Ti plate to simulate an herbarium specimen, which are always adhered to a cardboard sheet as illustrated in Fig. 3.

### Areal density modelling

Herbarium specimens can be classified as an ‘intermediate sample’ for the K-α line of elements with atomic numbers between 16 and 43 [13]. The intensities of these elements’ K-α line fluorescence in an intermediate sample are dependent on sample thickness and full matrix composition and increase with thickness increases until reaching a certain thickness that gives saturated intensity, called thick sample or critical thickness [35]. The fluorescence intensity of an element at a given thickness is defined as follows:

1 $${I}_{i}={I}_{i}^{thick}*\left(1-exp \left[-{\mu }_{i}\rho t\right]\right)$$

where, ${I}_{i}$—the intensity of *i*th element at given thickness, ${I}_{i}^{thick}$—the intensity of *i*th element in thick sample, $\rho$—density sample (g cm^−3^), $t$—sample thickness (cm), and ${\mu }_{i}$—total mass attenuation coefficient (cm^2^ g^−1^).

The relative intensity of *i*th element is a linear function of the concentration of the *i*th element based on the fundamental algorithm [51]. Because the relative intensity of *i*th element in an intermediate sample is thickness dependent, as illustrated in Fig. 1, it is crucial to correct the relative intensity for thickness variations.

Dynamic Analysis, the Fundamental Parameters approach, has been developed for nuclear microprobe and synchrotron-based XRF [43, 44]. The algorithm constructs a matrix transform to mimic a linear least-square fit to an XRF spectrum, in order to deconvolved the spectrum into fluorescence peaks for each element [46] as shown in Fig. 2. The advantages of Fundamental Parameters such as Dynamic Analysis, compared to the empirical calibrations, are that the Fundamental Parameters does not require standards, and can be used for other elements, while the empirical method needs a set of standards for each element and sample type [29, 35–37]. The SDD detector of the handheld XRF instrument is has about 150 eV resolution. As a consequence, overlapping peaks are indistinguishable spectroscopically, for example, a common peak overlap between Fe Kβ1 line (at 7.058 keV) with the Co Kα1 line (6.9303 keV). Nevertheless, Dynamic Analysis can separate overlapping peaks of Co and Fe shown in Fig. 2b. Therefore, the Dynamic Analysis approach is a powerful alternative to the built-in algorithm of a handheld XRF instrument while having complete control over the input parameters (as opposed to the ‘blackbox’ propriety software used by XRF manufacturers). The GeoPIXE is a software package that is based on the Dynamic Analysis method [47].

The major shortcoming of the Fundamental Parameters approach, such as in Dynamic Analysis, is that prior knowledge of sample areal density (g/cm^2^) is required [38, 39]. Many approaches to simultaneously determine the areal density of a sample and its elemental composition have been proposed, including adding an internal standard [40] or combining XRF and other techniques [41], however none of these methods compatible with herbarium XRF scanning. The areal density of a sample can also be determined using emission-transmission technique [42]. This requires three measurements: herbarium specimen, a substrate (pure thick element), and herbarium specimen on top of the substrate [35, 42]. If *I*_s_, *I*_t_, and *I*_ts_ refer to the Ti intensities from the sample alone, target alone, and the sample on top of the target, respectively, then the relationship between intensities and areal density can be expressed as follows:

2 $$\frac{{I}_{ts}-{I}_{s}}{{I}_{t}}=\mathrm{exp}\left[-\mu m\right]$$

where $m$, areal density (g/cm^2^), is the product of density $\rho$ multiplied by sample thickness $t$. Until now, all the herbarium XRF scanning conducting so far uses a 2 mm thick pure Ti plate [17–20], and Ti concentration in leaves is less than 34 µgg^−1^ or even less [49, 50], thus not producing significant Ti fluorescence. So, Eq. (2) can be simplified to:

3 $${I}_{ts}={I}_{t}\mathrm{exp}\left[-\mu m\right]$$

From Eq. (3) can be seen that the value of ${I}_{ts}$ relies upon the sample matrix because the value of ${I}_{t}$ is constant for all measurement. Equation (3) also infers that instead of three measurements (herbarium specimen, a 2 mm thick pure Ti plate, and a 2 mm thick pure Ti plate) under the specimen, one measurement of herbarium specimen on top of a 2 mm thick pure Ti plate could suffice to model the areal density. It is assumed that the variations in Ti concentrations are resulted from variations in areal density. Empirical models were built to quantify the variation and later could be used to predict the areal density of unknown sample. The procedure of establishing the empirical models for predicting areal density is as follows:

1. Each a 6 mm diameter leaf was placed at the geometric centre of the examination window and subsequently measured using the handheld XRF spectrometer. A stack of a sheet of cardboard, a 2 mm thick pure Ti plate, and a 2 mm thick pure Mo plate were placed below the leaf disc for the measurements.
2. The obtained XRF spectrum was processed using the Dynamic Analysis algorithm by providing an initial density 10.56 mg/cm^2^ corresponding to the mean areal density of the training dataset.
3. The Ti concentrations reported by the Dynamic Analysis algorithm with the initial density were plotted against Ti concentrations reported by the Dynamic Analysis algorithm with the measured areal density that were plotted also against the measured areal density.

### Validation of the proposed approach

The dataset was divided into two datasets, and the proportion of the training and test dataset is 80% (470 samples) and 20% (118 samples), following the Pareto principle. The performance of Dynamic Analysis was compared against the built-in algorithm and empirical calibration approach that used the training dataset to build the empirical model. The results of ICP-AES were used as the reference data, and mean absolute error was used to quantify the magnitude of the errors generated by each approach compared to the ICP-AES results. Also, ratioing the elemental concentration of each approach to ICP-AES was performed to evaluate the direction of the errors whether underestimated or overestimated.

### Dynamic analysis in GeoPIXE

Three batch processing were conducted in GeoPIXE. The first batch aimed to obtain the instrument related parameters and was achieved by processing three certified thin films (Ti, Au, and Sn). Because the parameters of the samples (the composition and thickness of the metal films) were known, the instrument related parameters in GeoPIXE were adjusted so that the concentration of the films is 100%. The second batch processing was designed to establish a procedure for predicting the areal density of an unknown sample. The last batch was performed on the test dataset based on the parameters and procedure obtained from the first and second batch processing. During the last batch, the continuum background of the spectrum is also corrected and estimated for fluctuated and low counts [52]. Then, the limit of detection is defined as following, 3.29 ${\sigma }_{b}$, where ${\sigma }_{b}$ is the background standard deviation [53]. The limit of detection for each spectrum differs due to differences in matrix compositions and physical properties, and thus, the limit of detection are the mean of the detection limit. The average limits of detection for K, Ca, Mn, Fe, Co, Ni, and Zn were 2936 µg g^−1^, 1829 µg g^−1^, 154 µg g^−1^, 127 µg g^−1^, 104 µg g^−1^, 134 µg g^−1^, and 101 µg g^−1^, respectively.

### ICP-AES analysis

The weight of all samples was recorded and the samples were pre-digested in 2 mL HNO_3_ (70%) for 48 h. After that, the samples were fully acid digested for 45 min in a microwave oven (Milestone Start D) using the following program rise to 80 °C within 10 min, rise to 135 °C in the next 15 min, hold for 20 min at 135 °C, and cool down for 30 min. The samples were then brought to volume (10 mL0 and analysed with an ICP-AES (Thermo Scientific iCAP 7400) instrument for natrium, magnesium, aluminium, phosphor, sulphur, K, Ca, chromium, Mn, Fe, Co, Ni, Cu, Zn, As, selenium, cadmium, and thulium in axial or radial modes depending on the expected analyte concentration. A 4-point calibration was used to calibrate all elements and to compensate for matrix-based effects, yttrium was used as an in-line internal addition standardization. Standard Reference Material (NIST Apple Leaves 1515), internal reference materials, certified reference material (Sigma-Aldrich Periodic Table mix 1 for ICP TraceCERT^®^), and matrix blanks were measured as quality controls. The results of ICP-AES analysis were used here as a reference for the XRF data calibrations.

### Statistical analysis

R version 3.6.2 was used for statistical analysis. A non-linear least square, nls, function which is the built-in function of R was used to fit an empirical model into the scatterplot between Ti concentration and areal density data. The “Metrics” package was used for calculating the mean absolute error of each prediction, and the “agricolae” package was used for conducting the non-parametric, Kruskal–Wallis, test. Meanwhile, the “ggplot” package was used for producing the charts.

### Spatial variation analysis

Because herbarium specimens were measured in situ, it is important to assess the spatial variability of elemental concentrations across the leaf. In this case, twenty three leaves were scanned using a custom-built µXRF instrument at the Centre for Microscopy and Microanalysis, the University of Queensland, Brisbane, Australia to map the elemental concentrations in the whole leaves. The instrument has a 50 kV (1000 μA) Mo-target source that generates 17.4 keV X-rays fitted with polycapillary focussing optics to 25 μm. The leaf specimens were placed between two sheets of 6 μm Ultralene thin film stretched over a Perspex frame magnetically attached to the sample platform of the instrument and measured at atmospheric temperature (~ 20 °C). The results are multi-band 'elemental' images and each band represents a discrete energy (and thus element). The multi-band images were processed in GeoPIXE producing elemental maps of different elements (K, Ca, Mn, Fe, Co, Ni, Zn, and Se) as shown in Fig. 9a. These elemental maps were further processed in QGIS 3.16 to segment each image into the specimen and the background (Fig. 9b). It was classified by manually setting the threshold between the specimen and the background. Three circles with a diameter of 6 mm were drawn at the apex, lamina, and base to simulate three measurements using a portable XRF instrument (Fig. 9c). The average concentration of each element per specimen was calculated (green area in Fig. 9b) and compared to the average concentration of the three spots shown in Fig. 9c. Table 3 lists specimens scanned using the µXRF instrument.

**Table 3:** List of specimens scanned using the µXRF instrument. The letter ‘X’ indicates elemental concentration image available for the corresponding specimen

Plant species	K	Ca	Mn	Fe	Co	Ni	Zn	Se
*Berkheya coddii*	X	X	X			X		
*Gossia bidwillii*	X	X	X					
*Camellia sinensis*	X	X	X					
*Camellia sinensis*	X	X	X					
*Coelospermum decipiens*	X	X	X	X				X
*Coelospermum decipiens*	X	X						X
*Coelospermum decipiens*	X	X						X
*Coelospermum decipiens*	X	X						X
*Coelospermum decipiens*	X	X						X
*Crotalaria novo-hollandiae*	X	X		X	X	X	X	
*Crotalaria novo-hollandiae*	X	X		X	X	X	X	
*Crotalaria novo-hollandiae*	X	X		X	X	X	X	
*Crotalaria novo-hollandiae*	X	X		X	X	X	X	
*Crotalaria novo-hollandiae*	X	X		X	X	X	X	
*Crotalaria novo-hollandiae*	X	X		X	X	X	X	
*Crotalaria novo-hollandiae*	X	X	X	X			X	
*Crotalaria novo-hollandiae*	X	X	X	X			X	
*Crotalaria novo-hollandiae*	X			X			X	
*Crotalaria novo-hollandiae*	X			X			X	
*Crotalaria novo-hollandiae*	X			X			X	
*Crotalaria novo-hollandiae*	X			X			X	
*Crotalaria novo-hollandiae*	X	X					X	
*Crotalaria novo-hollandiae*	X	X					X	

## Supplementary Information

**Additional file 1: Table S1. **The results of XRF measurements on the spiked samples. **Table S2. **Relative standard deviation based on data provided in Table S1. **Table S3. **The parameters of X-ray spectrum fit used during this study. **Table S4. **The parameters related to the detector obtained after calibration based on the thin films. **Table S5. **The parameters using during the yield calculation of Ti pure thick and thin film. **Table S6**. Source setup in GeoPIXE. **Figure S1. **The result of fitting model in GeoPIXE into an XRF spectrum of Mn hyperaccumulator dry leaf. **Figure S2. **The result of fitting model in GeoPIXE into an XRF spectrum of Co hyperaccumulator dry leaf. **Figure S3. **The result of fitting model in GeoPIXE into an XRF spectrum of Ni hyperaccumulator dry leaf. **Figure S4. **The result of fitting model in GeoPIXE into an XRF spectrum of Zn hyperaccumulator dry leaf.

## Data Availability

The data underlying this article will be shared on reasonable request to the corresponding author.

## References

[1] van der Ent A, Echevarria G, Pollard AJ, Erskine PD (2019). X-Ray fluorescence ionomics of herbarium collections. Sci Rep.

[2] Salt DE, Baxter I, Lahner B (2008). Ionomics and the study of the plant ionome. Annu Rev Plant Biol.

[3] Baxter IR, Vitek O, Lahner B, Muthukumar B, Borghi M, Morrissey J (2008). The leaf ionome as a multivariable system to detect a plant’s physiological status. Proc Natl Acad Sci USA.

[4] Peñuelas J, Fernández-Martíne M, Ciais P, Jou D, Piao S, Obersteiner M, Vicca S, Janssens IA, Sardans J (2019). The bioelements, the elementome, and the biogeochemical niche. Ecology.

[5] van der Ent A, Ocenar A, Tisserand R, Sugau JB, Echevarria G, Erskine PD (2019). Herbarium X-ray fluorescence screening for nickel, cobalt and manganese hyperaccumulator plants in the flora of Sabah (Malaysia, Borneo Island). J Geochemical Explor.

[6] Markowicz A, Van Grieken R, Markowicz A (2001). X-ray physics. Handb X-ray spectrom.

[7] Potts PJ (2008). Chapter 1. Introduction, analytical instrumentation and application overview portable X-ray fluoresc spectrom.

[8] Kanngießer B, Haschke M, Simionovici A, Chevallier P, Streli C, Wobrauschek P, Beckhoff B, Kanngießer B, Langhoff N, Wedell R, Wolff H (2005). Methodological developments and applications. Handb pract X-Ray fluoresc anal.

[9] Antonangelo J, Zhang H (2021). Soil and plant nutrient analysis with a portable XRF probe using a single calibration. Agronomy.

[10] Reidinger S, Ramsey MH, Hartley SE (2012). Rapid and accurate analyses of silicon and phosphorus in plants using a portable X-ray fluorescence spectrometer. New Phytol.

[11] Towett EK, Shepherd KD, Lee DB (2016). Plant elemental composition and portable X-ray fluorescence (pXRF) spectroscopy: quantification under different analytical parameters. X-Ray Spectrom.

[12] Zhou S, Weindorf DC, Cheng Q, Yang B, Yuan Z, Chakraborty S (2020). Elemental assessment of vegetation via portable X-ray fluorescence: sample preparation and methodological considerations. Spectrochim Acta Part B At Spectrosc.

[13] Purwadi I, Gei V, Echevarria G, Erskine PD, Mesjasz-Przybyłowicz J, Przybyłowicz WJ, van der Ent A, Baker AJM, Echevarria G, Simonnot M-O, Morel JL (2021). Tools for the discovery of hyperaccumulator plant species in the field and in the herbarium. Agromining farming met.

[14] Reeves RD (2003). Tropical hyperaccumulators of metals and their potential for phytoextraction. Plant Soil.

[15] Baker JM, Brooks RR (1989). Terrestrial higher plants which hyperaccumulate metallic elements—a review of their distribution, ecology and phytochemistry. Biorecovery..

[16] van der Ent A, Baker AJM, Reeves RD, Pollard AJ, Schat H (2013). Hyperaccumulators of metal and metalloid trace elements: facts and fiction. Plant Soil.

[17] Brooks RR, Brooks RR (1998). Plants that hyperaccumulate heavy metals: their role in phytoremediation, microbiology, archaeology, mineral exploration and phytomining.

[18] Wulff AS, Hollingsworth PM, Ahrends A, Jaffré T, Veillon JM, L’Huillier L (2013). Conservation priorities in a biodiversity hotspot: analysis of narrow endemic plant species in New Caledonia. PLoS ONE..

[19] Erskine P, van der Ent A, Fletcher A (2012). Sustaining metal-loving plants in mining regions. Science.

[20] Whiting SN, Reeves RD, Richards D, Johnson MS, Cooke JA, Malaisse F (2010). Research priorities for conservation of metallophyte biodiversity and their potential for restoration and site remediation. Restor Ecol.

[21] Guerin GR (2013). The value of herbaria to diverse collections-based research. Australas Syst Bot Soc Newsl.

[22] Nkrumah PN, Echevarria G, Erskine PD, van der Ent A (2018). Nickel hyperaccumulation in *Antidesma montis-silam*: from herbarium discovery to collection in the native habitat. Ecol Res.

[23] Do C, Abubakari F, Remigio AC, Brown GK, Casey LW, Burtet-Sarramegna V (2020). A preliminary survey of nickel, manganese and zinc (hyper)accumulation in the flora of Papua New Guinea from herbarium X-ray fluorescence scanning. Chemoecology..

[24] Gei V, Isnard S, Erskine PD, Echevarria G, Fogliani B, Jaffré T (2020). A systematic assessment of the occurrence of trace element hyperaccumulation in the flora of New Caledonia. Bot J Linn Soc..

[25] McCartha GL, Taylor CM, van der Ent A, Echevarria G, Navarrete Gutiérrez DM, Pollard AJ (2019). Phylogenetic and geographic distribution of nickel hyperaccumulation in neotropical Psychotria. Am J Bot.

[26] ThermoFisher. Portable XRF calibrations in well site geochemistry . 2015. https://www.thermofisher.com/blog/mining/portable-xrf-calibrations-in-well-site-geochemistry/. Accessed 2 May 2020.

[27] Bruker. XRF data differences: quantitative, semi-quantitative, and qualitative data. 2018. https://www.bruker.com/products/x-ray-diffraction-and-elemental-analysis/handheld-xrf/xrf-data-primer-quantitative-semi-quantitative-qualitative.html. Accessed 2 May 2020.

[28] Sherman J (1955). The theoretical derivation of fluorescent X-ray intensities from mixtures. Spectrochim Acta.

[29] Rousseau R (2013). How to apply the fundamental parameters method to the quantitative X-ray fluorescence analysis of geological materials. J Geosci Geomatics.

[30] Markowicz A (2011). An overview of quantification methods in energy-dispersive X-ray fluorescence analysis. Pramana.

[31] Bosco GL (2013). Development and application of portable, hand-held X-ray fluorescence spectrometers. TrAC Trends Anal Chem.

[32] Nielson KK (1977). Matrix corrections for energy dispersive X-ray fluorescence analysis of environmental samples with coherent/incoherent scattered X-rays. Anal Chem.

[33] Kenna TC, Nitsche FO, Herron MM, Mailloux BJ, Peteet D, Sritrairat S (2011). Evaluation and calibration of a field portable X-Ray fluorescence spectrometer for quantitative analysis of siliciclastic soils and sediments. J Anal At Spectrom.

[34] Rousseau RM, Willis JP, Duncan AR (1996). Practical XRF calibration procedures for major and trace elements. X-Ray Spectrom.

[35] Sitko R (2009). Quantitative X-ray fluorescence analysis of samples of less than “infinite thickness”: difficulties and possibilities. Spectrochim Acta Part B At Spectrosc.

[36] Sitko R, Zawisza B, Czaja M (2005). Fundamental parameters method for determination of rare earth elements in apatites by wavelength-dispersive X-ray fluorescence spectrometry. J Anal At Spectrom.

[37] Rousseau RM (2006). Corrections for matrix effects in X-ray fluorescence analysis—a tutorial. Spectrochim Acta Part B At Spectrosc.

[38] Wróbel PM, Bała S, Czyzycki M, Golasik M, Librowski T, Ostachowicz B (2017). Combined micro-XRF and TXRF methodology for quantitative elemental imaging of tissue samples. Talanta.

[39] Szczerbowska-Boruchowska M (2012). Sample thickness considerations for quantitative X-ray fluorescence analysis of the soft and skeletal tissues of the human body—theoretical evaluation and experimental validation. X-Ray Spectrom.

[40] Wȩgrzynek D, Hołyńska B, Ostachowicz B (1998). A comparison of the performance of a fundamental parameter method for analysis of total reflection X-ray fluorescence spectra and determination of trace elements, versus an empirical quantification procedure. Spectrochim Acta Part B At Spectrosc.

[41] Lagomarsino S, Iotti S, Farruggia G, Cedola A, Trapani V, Fratini M (2011). Intracellular concentration map of magnesium in whole cells by combined use of X-ray fluorescence microscopy and atomic force microscopy. Spectrochim Acta Part B At Spectrosc.

[42] Wegrzynek D, Markowicz A, Chinea-Cano E, Bamford S (2003). Evaluation of the uncertainty of element determination using the energy-dispersive x-ray fluorescence technique and the emission-transmission method. X-Ray Spectrom.

[43] Ryan CG, Etschmann BE, Vogt S, Maser J, Harland CL, Van Achterbergh E (2005). Nuclear microprobe—synchrotron synergy: towards integrated quantitative real-time elemental imaging using PIXE and SXRF. Nucl Instruments Methods Phys Res Sect B Beam Interact Mater Atoms.

[44] Ryan CG (2000). Quantitative trace element imaging using PIXE and the nuclear microprobe. Int J Imaging Syst Technol.

[45] Criss JW, Birks LS (1968). Calculation methods for fluorescent x-ray spectrometry. Empirical coefficients versus fundamental parameters. Anal Chem..

[46] Ryan CG, Laird JS, Fisher LA, Kirkham R, Moorhead GF (2015). Improved dynamic analysis method for quantitative PIXE and SXRF element imaging of complex materials. Nucl Instruments Methods Phys Res Sect B Beam Interact Mater Atoms.

[47] van der Ent A, Przybyłowicz WJ, de Jonge MD, Harris HH, Ryan CG, Tylko G (2018). X-ray elemental mapping techniques for elucidating the ecophysiology of hyperaccumulator plants. New Phytol.

[48] Markowicz AA, Potts PJ, West M (2008). Chapter 2. Quantification and correction procedures. Portable X-ray fluoresc spectrom.

[49] Cary EE, Kubota J (1990). Chromium concentration in plants : effects of soil chromium concentration and tissue contamination by soil. J Agric Food Chem.

[50] Tlustoš P, Cígler P, Hrubý M, Kužel S, Száková J, Balík J (2011). The role of titanium in biomass production and its influence on essential elements’ contents in field growing crops. Plant Soil Environ.

[51] Rousseao RM (1984). Fundamental algorithm between concentration and intensity in XRF analysis. X-Ray Spectrom.

[52] Ryan CG, Clayton E, Griffin WL, Sie SH, Cousens DR (1988). SNIP, a statistics-sensitive background treatment for the quantitative analysis of PIXE spectra in geoscience applications. Nucl Inst Methods Phys Res B.

[53] Currie LA (1968). Limits for qualitative detection and quantitative determination: application to radiochemistry. Anal Chem.

