# Supplementary material for: X-ray fluorescence spectroscopy (XRF) for metallome analysis of herbarium specimens
Source: Plant Methods. 2022 Dec 19;18:139. doi: 10.1186/s13007-022-00958-z (PMC9761992; doi:10.1186/s13007-022-00958-z)
Supplement: Supplementary file 1 — Additional file 1: Table S1. The results of XRF measurements on the spiked samples. Table S2. Relative standard deviation based on data provided in Table S1. Table S3. The parameters of X-ray spectrum fit used during this study. Table S4. The parameters related to the detector obtained after calibration based on the thin films. Table S5. The parameters using during the yield calculation of Ti pure thick and thin film. Table S6. Source setup in GeoPIXE. Figure S1. The result of fitting model in GeoPIXE into an XRF spectrum of Mn hyperaccumulator dry leaf. Figure S2. The result of fitting model in GeoPIXE into an XRF spectrum of Co hyperaccumulator dry leaf. Figure S3. The result of fitting model in GeoPIXE into an XRF spectrum of Ni hyperaccumulator dry leaf. Figure S4. The result of fitting model in GeoPIXE into an XRF spectrum of Zn hyperaccumulator dry leaf. [file 13007_2022_958_MOESM1_ESM.pdf]

Additional file 1

X-ray fluorescence spectroscopy (XRF) for metallome analysis of herbarium specimens

Imam Purwadi[1], Lachlan W. Casey[2], Chris G. Ryan[3], Peter D. Erskine[1], Antony van der Ent[1,4*]

[1]Centre for Mined Land Rehabilitation, Sustainable Minerals Institute,
The University of Queensland, Queensland, Australia.

[2]Centre for Microscopy and Microanalysis, The University of Queensland, Australia.

[3]CSIRO, Mineral Resources, Australia.

[4]Laboratoire Sols et Environnement, INRAE, Université de Lorraine, France.

*Corresponding author: A. van der Ent (a.vanderent@uq.edu.au). Centre for Mined Land Rehabilitation, Sustainable Minerals Institute, The University of Queensland, Brisbane QLD 4072, Australia.

Procedure data acquisition shown in Figure 1

In illustrating the effect of thickness on the XRF readings, spiked samples are prepared as follows:

1. *Elaeocarpus eumundii* fresh leaves were collected from the vicinity of our laboratory. This species was chosen due to its abundance.
2. *Elaeocarpus eumundii* fresh leaves were oven-dried at 60ºC for 3 days.
3. The dried leaves were pulverized and sieved using a 0.25µm fine mesh sieve to attain uniform particle size.
4. The pulverized leaves divided into several portions. Each of four portions were spiked with 1000 $\mu g\ g^{-1}$ of Mn, Co, Cu, or Zn in the form of MnO_2, Co_3O_4, CuO, and ZnO, respectively. Each of two portions were spiked with 10000 $\mu g\ g^{-1}$ of S or Ca in the form of S and CaO, respectively.
5. The mixtures of the pulverized leaves and spiked agents were shaken using the end-over-end shaker for 24h.
6. From each mixture, three replicate weights of 0.12g, 0.2g, 1.5g, and 3.0g for each of the mixtures was prepared. These are carefully transferred into the pre-assembled XRF cups and shaking gently to ensure all parts of the cup was completely covered with sample. A 25mm filter paper was placed next to it before a pure cellulose powder is added as 'backing agent'.
7. To determine the effectiveness of each of the time measurements, each sample cup was measured with the instrument for 60s using Soils calibration modes. The results of XRF readings are provided in the Table S1.

Table S1. The results of XRF measurements on the spiked samples.

No	Element	Replicate	Weight (g)	XRF Concentration (µg g^{-1})	No	Element	Replicate	Weight (g)	XRF Concentration (µg g^{-1})
1	S	1	0.12	2642	37	Co	1	0.12	510.1
2	S	2	0.12	2215	38	Co	2	0.12	560.2
3	S	3	0.12	2667	39	Co	3	0.12	429.1
4	S	1	0.2	2809	40	Co	1	0.2	524.1
5	S	2	0.2	2528	41	Co	2	0.2	666.3
6	S	3	0.2	2892	42	Co	3	0.2	647.2
7	S	1	1.5	2726	43	Co	1	1.5	1295.9
8	S	2	1.5	3038	44	Co	2	1.5	1367.4
9	S	3	1.5	2419	45	Co	3	1.5	1358.5
10	S	1	3	2679	46	Co	1	3	1300.6
11	S	2	3	2524	47	Co	2	3	1326.8
12	S	3	3	2768	48	Co	3	3	1342.2
13	Ca	1	0.12	33412	49	Cu	1	0.12	343
14	Ca	2	0.12	29004	50	Cu	2	0.12	403
15	Ca	3	0.12	32805	51	Cu	3	0.12	482
16	Ca	1	0.2	36259	52	Cu	1	0.2	344
17	Ca	2	0.2	35252	53	Cu	2	0.2	649
18	Ca	3	0.2	36280	54	Cu	3	0.2	450
19	Ca	1	1.5	35423	55	Cu	1	1.5	1221
20	Ca	2	1.5	35477	56	Cu	2	1.5	1254
21	Ca	3	1.5	35560	57	Cu	3	1.5	1222
22	Ca	1	3	34580	58	Cu	1	3	1241
23	Ca	2	3	35829	59	Cu	2	3	1247
24	Ca	3	3	34915	60	Cu	3	3	1223
25	Mn	1	0.12	414	61	Zn	1	0.12	364
26	Mn	2	0.12	462	62	Zn	2	0.12	381
27	Mn	3	0.12	391	63	Zn	3	0.12	206
28	Mn	1	0.2	323	64	Zn	1	0.2	257
29	Mn	2	0.2	558	65	Zn	2	0.2	399
30	Mn	3	0.2	514	66	Zn	3	0.2	665
31	Mn	1	1.5	688	67	Zn	1	1.5	1282
32	Mn	2	1.5	705	68	Zn	2	1.5	1315
33	Mn	3	1.5	628	69	Zn	3	1.5	1288
34	Mn	1	3	612	70	Zn	1	3	1278
35	Mn	2	3	574	71	Zn	3	3	1333
36	Mn	3	3	687	72	Zn	3	3	1339

Table S2. Relative standard deviation based on data provided in Table S1.

Element	Duration (second)	Concentration (μg g^{-1})	Standard deviation	Average	Relative Standard Deviation
Ca	30.0	34657.5			
Ca	30.0	35096.9	237.3	34825.3	0.7%
Ca	30.0	34721.7			
Ca	60.0	34579.8			
Ca	60.0	35829.3	646.8	35107.9	1.8%
Ca	60.0	34914.5			
Ca	120.0	35864.6			
Ca	120.0	35761.3	73.1	35813.0	0.2%
Ca	120.0				
Co	30.0	1305.7			
Co	30.0	1301.4	20.6	1315.4	1.6%
Co	30.0	1339.1			
Co	60.0	1300.6			
Co	60.0	1326.8	21.0	1323.2	1.6%
Co	60.0	1342.2			
Co	120.0	1292.5			
Co	120.0	1305.1	26.8	1313.9	2.0%
Co	120.0	1344.0			
Cu	30.0	1239.2			
Cu	30.0	1243.6	10.3	1235.6	0.8%
Cu	30.0	1224.0			
Cu	60.0	1240.7			
Cu	60.0	1247.4	12.8	1237.0	1.0%
Cu	60.0	1222.7			
Cu	120.0	1237.9			
Cu	120.0	1221.6	9.2	1227.3	0.7%
Cu	120.0	1222.5			
Mn	30.0	619.8			
Mn	30.0	617.8	24.1	604.9	4.0%
Mn	30.0	577.0			
Mn	60.0	611.8			
Mn	60.0	574.3	57.6	624.5	9.2%
Mn	60.0	687.4			
Mn	120.0	698.0			
Mn	120.0	667.1	54.2	652.5	8.3%
Mn	120.0	592.5			
Zn	30.0	1299.0			
Zn	30.0	1329.6	15.4	1313.4	1.2%
Zn	30.0	1311.7			
Zn	60.0	1278.2			
Zn	60.0	1333.4	33.4	1316.7	2.5%
Zn	60.0	1338.5			
Zn	120.0	1281.3			
Zn	120.0	1315.6	29.7	1312.5	2.3%
Zn	120.0	1340.5			

Table S3. The parameters of X-ray spectrum fit used during this study

Element<br>Ti, Sn, Au (For thin film)<br>Ar, K, Ca, Sc, Ti, Cr, Mn, Fe, Co, Ni, Cu, Zn, Br, Sr (for samples)	
Energy Range	1-30
Det	See Table S3
Q	3
Filter	Air 15mm + 4 micrometre thick Ultralene
Yield	See Table S4
Advanced tab<br>Background: SNIP	8

Table S4. The parameters related to the detector obtained after calibration based on the thin films

Material	Si (Si(Li) or Si
Model	X-ray detector model
Diameter	5.6419
Distance	1
Thickness	1.4
Area	25
Solid Angle	24620.2
Resolution	0.15
Tilt Angle	10
Shape	Round, Circular
Array	Single detector
Scale factor of Cohen et al	Off
Volume solid-angle correction	On
Absorber Layer Definitions	
#absorbers	2
# absorber 1	
Thick	5 microns
Density	1.803
Formula	Be
Special	Plain filter
# absorber 2	
Thick	100 microns
Density	4.52
Formula	Ti
Special	Pinhole filter
Solid-angle ratio	1.2

Table S5. The parameters using during the yield calculation of Ti pure thick and thin film.

**Beam Particle**	
Photons	Continuous
Source	See Table S5
Energy Range	
E min	2
E max	48
**Detector**	
Theta	90
Phi	0
Target	
Alpha	-45
Beta	0
**Target Layer Selection**	
Layers	1
Unknown	1
# Define layer 1<br>Areal density<br>Density<br>Formula	 See Figure 4<br>0.9<br>$C_6H_{10}O_5$

Table S6. Source setup in GeoPIXE.

**Anode**	
Formula	Ag
**Source Parameters**	
Volts	50
Power	0.24
Angle in	45
Angle out	45
Spot	0.001
Omega	2000
Inline optics	None
**Filter**	
#filters	2
#filter 1<br>Thick<br>Density<br>Formula<br>Special	 101.6 micron<br>7.867<br>Fe<br>Plain filter
#filter 2<br>Thick<br>Density<br>Formula<br>Special	 50.8 micron<br>10.47<br>Ag<br>Plain filter

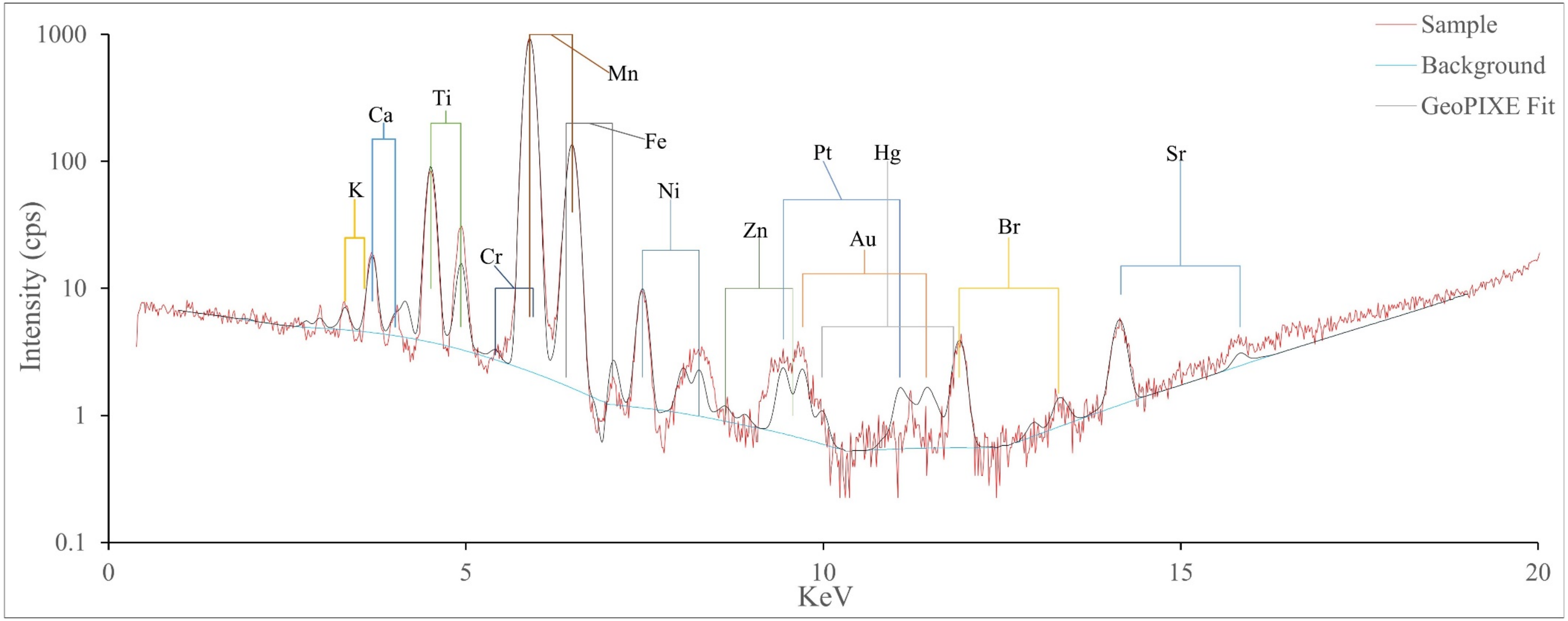

Figure S1. The result of fitting model in GeoPIXE into an XRF spectrum of Mn hyperaccumulator dry leaf.

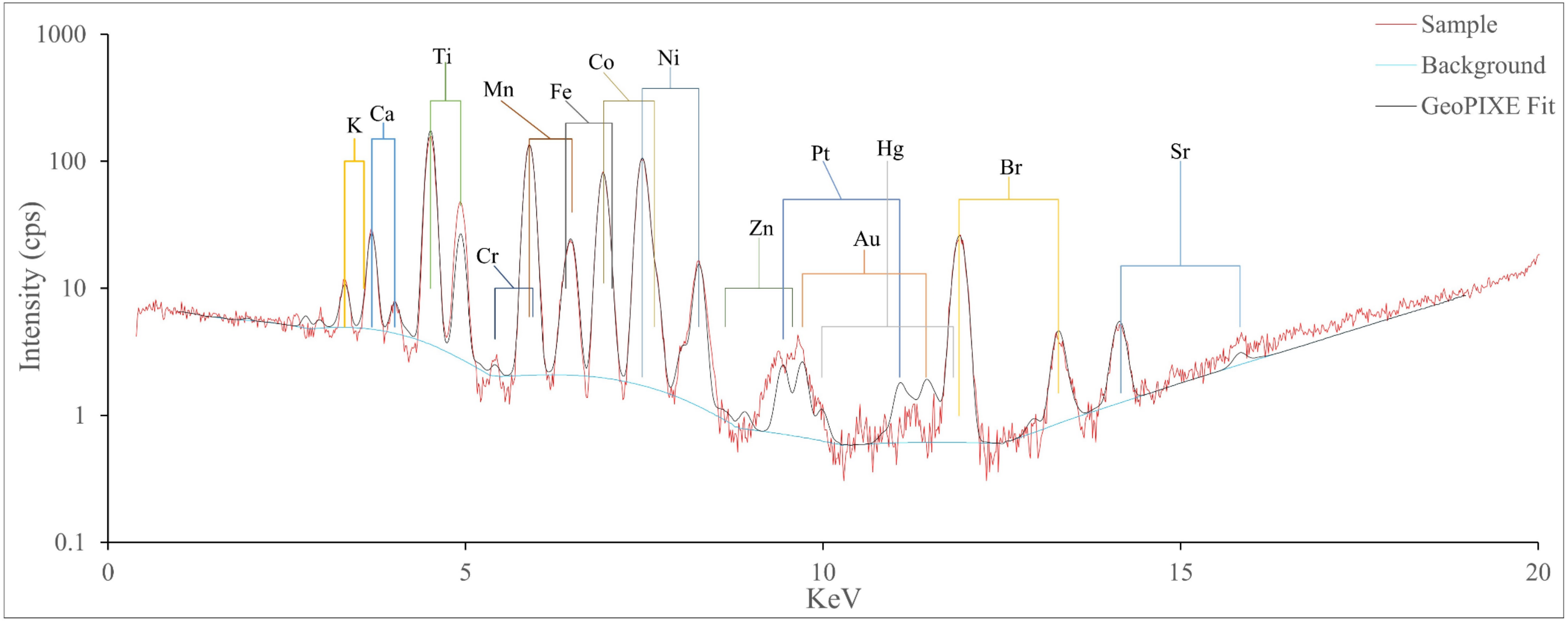

Figure S2. The result of fitting model in GeoPIXE into an XRF spectrum of Co hyperaccumulator dry leaf.

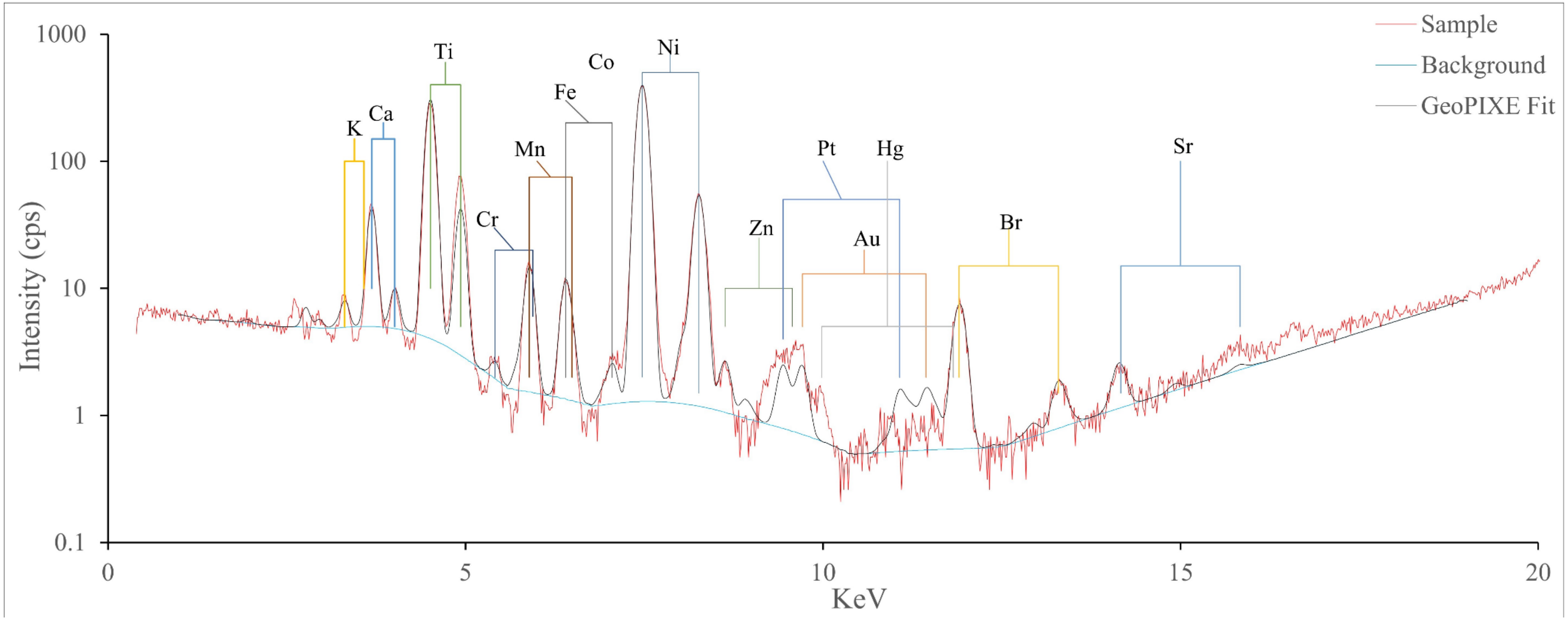

Figure S3. The result of fitting model in GeoPIXE into an XRF spectrum of Ni hyperaccumulator dry leaf.

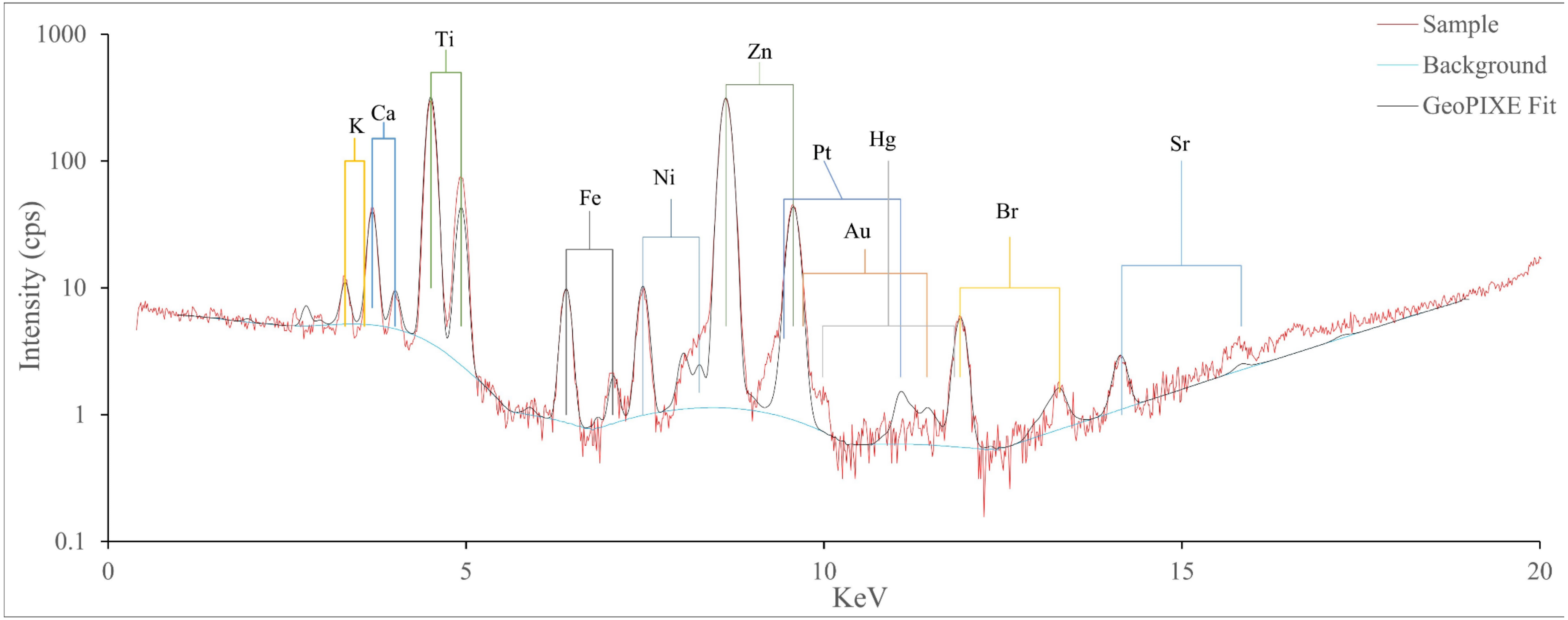

Figure S4. The result of fitting model in GeoPIXE into an XRF spectrum of Zn hyperaccumulator dry leaf.
